# Forecasting viral evolution from phylogenetic trees

**DOI:** 10.64898/2026.08.27.741102

**Published:** 2026-09-05

**Authors:** Ivan Specht, Soyoon Park, Seyone Chithrananda, Claudia L. Driscoll, Garyk Brixi, Julia A. Palacios, Brian L. Hie

**Affiliations:** 1 Stanford University, Stanford, CA, USA; 2 Arc Institute, Palo Alto, CA, USA.

## Abstract

Viral mutation forecasting plays a key role in pandemic preparedness by enabling researchers to anticipate novel variants and design proactive interventions. Evolutionary histories, represented as phylogenetic trees, offer key insights into the emergence of past and present strains, yet their role in predicting future sequence changes remains largely unexplored. We introduce antiGen, a machine learning model that forecasts the evolutionary future of viruses by learning from their evolutionary past. antiGen achieves state-of-the-art performance for predicting mutations to the SARS-CoV-2 spike protein, anticipating never-before-seen mutations and mutations that emerge years after the model’s training window. antiGen-forecasted spike mutations also retain pseudoviral infectivity *in vitro*. Moreover, antiGen demonstrates leading predictive performance on surface proteins of influenza virus, respiratory syncytial virus, and dengue virus despite far less available sequencing data. Viral evolution models that explicitly learn from phylogenetic structure offer a valuable resource for applications ranging from epidemiological modeling to therapeutic development.

## Introduction

The evolution of viral diseases poses a challenge to effective outbreak response (Grubaugh et al., 2019). As viruses spread through populations, they accrue mutations that may render them more transmissible, more harmful, and more likely to evade immunity (Harvey et al., 2021). Mutation prediction models can help mitigate these risks by providing targets for vaccines and other pharmaceutical interventions, allowing them to remain robust to future evolutionary changes (Łuksza and Lässig, 2014; Fraser et al., 2021).

Previous studies have proposed experimental and computational methods to tackle the variant prediction problem. Deep mutational scanning (DMS), an experimental procedure that measures the effect of every single amino acid substitution on the fitness of a protein (Starr et al., 2022; Taylor and Starr, 2026), is often considered a gold standard for quantifying these effects and is widely used for benchmarking computational methods (Notin et al., 2023). Nonetheless, DMS experiments are limited in their applicability to the viral mutation forecasting problem: they measure fitness independently of mutational accessibility (e.g., the number of nucleotide changes needed for an amino acid substitution), and cannot account for unavoidable discrepancies between experimental conditions and real-world epidemic propagation (Mehrotra et al., 2025). Moreover, they are time-intensive, which can pose an issue amid rapidly changing epidemiological conditions that demand immediate response.

Computational methods for mutation prediction have shown promise in recent years, but considerable room for improvement remains in anticipating future viral variants. Protein language models (Lin et al., 2023; Hie et al., 2021), trained on vast databases of amino acid sequences, are useful for biological tasks ranging from functional annotation to structure prediction, but struggle on pandemic variant prediction without fine-tuning (Ito et al., 2025). The current leading computational approaches, such as EVE (Fraser et al., 2021) and derived models including EVEscape (Thadani et al., 2023), use neural networks trained on multiple sequence alignments (MSAs) for the viral protein of interest to better guide predictions. While including an MSA as input substantially improves mutation forecasts, these models still underperform basic statistical or biochemical predictors on several evaluations.

An intuitive starting point for modeling future mutations is to learn from a database of relevant past ones. The ancestral history of a set of samples is often represented as a phylogenetic tree, which encodes inferred ancestral relationships among taxa and thus enables reconstruction of the evolutionary changes that occurred between a sequence and its parent. Previous work has demonstrated that “parent-child pairs” extracted from a phylogeny can be used to learn the fitness effects of antibody mutations (Matsen et al., 2026). Moreover, the related concept of “phylogenetic cherries” (minimally diverged sequence pairs) from phylogenies across the tree of life has shown promise as a source of training data for protein language models (Koehl et al., 2026). Phylogeny-based training for viral mutation forecasting, however, has remained unexplored.

We introduce antiGen, a machine learning model trained directly on phylogenetic trees that delivers highly accurate future variant forecasts based on available genomic surveillance data (Figure 1). The key conceptual advance behind our work is to train on the inferred evolutionary changes between neighboring genotypes on the phylogeny, in contrast to protein language models and other methods that train on the sequences themselves. This approach aligns the training objective with the prediction task and remains robust to overrepresentation of certain genotypes in training data, which is a known limitation of protein language models (Ding and Steinhardt, 2024; Gordon et al., 2025).

First, given the challenge of inferring phylogenetic trees on large-scale surveillance data, we developed a high-performance computational pipeline for constructing pandemic phylogenies with millions of leaves, taking inspiration from the UShER algorithm (Turakhia et al., 2021). We then partitioned these trees into training and testing examples, each of which consists of a genotype from some point in the evolutionary past together with the set of single amino acid changes inferred to have altered that genotype. Based on these examples, we trained antiGen to learn the likelihood of each possible amino acid substitution relative to a given starting sequence, testing various architectures including a small Transformer trained from randomly initialized parameters as well as fine-tuned versions of existing models.

We demonstrate antiGen’s superior predictive power for future SARS-CoV-2 spike protein mutations based on both computational and experimental validation. Computationally, antiGen not only outperformed the current state of the art on predicting the most likely next mutations for a query sequence, but also outperformed existing methods on inferring previously unseen mutations and mutations that appear far later in time than the query. In some scenarios, such as data-limited regimes, combining antiGen with existing models further improved performance. Experimentally, most antiGen-forecasted SARS-CoV-2 spike mutants—including previously unseen ones—retained pseudoviral infectivity, supporting the functional plausibility of proposed mutations. Moreover, antiGen accurately forecasted influenza, respiratory syncytial virus (RSV), and dengue virus mutations, indicating its applicability to a diverse set of endemic viruses. By providing reliable predictions of future evolutionary changes, our work could serve as the basis for proactive vaccine and therapeutic design.

## Results

### Data generation and training

Training antiGen on inferred mutations in the evolutionary history of a sample first requires a method for obtaining large-scale phylogenetic trees. To do so, we designed an efficient program that accepts a small ‘guide tree’ with inferred ancestral states and inner node times (such as those available on Nextstrain (Hadfield et al., 2018)), together with a large protein MSA, and iteratively attaches each sequence in the MSA to the guide tree at the location that introduces the fewest new mutations (i.e., parsimonious placement). Our approach for tree construction scales to databases containing millions of sequences. Having constructed a large phylogenetic tree, we defined our ‘training tree’ to be the subtree consisting of all nodes and edges ancestral to the samples collected before a given cutoff date; the remainder we call the ‘testing tree(s).’ Finally, we extracted each example, defined as an inferred ancestral genotype together with the inferred single amino acid substitutions relative to that genotype (Figure 1B; Methods).

We trained antiGen to learn a joint probability distribution over the position along the genome and the new amino acid state involved in a substitution, conditional on the starting sequence. To infer the parameters governing this distribution, we defined our loss function to be the negative log likelihood of the true mutations observed across the training examples, normalized by the total number of mutations (Equation 1). Minimizing the loss function is then equivalent to computing the maximum likelihood estimate of these parameters (Methods).

antiGen’s architecture consists of a small, randomly initialized Transformer (Vaswani et al., 2017). The model encodes each amino acid via a learned token embedding summed with a learned absolute positional embedding, processes the sequence with two standard Transformer encoder layers, and produces per-position logits over the amino acid vocabulary through a final linear projection. While the exact model size varies with the length of the protein of interest (range: 465,558–565,142 trainable parameters for proteins studied in this paper), antiGen consistently trained in under 3 minutes on a single NVIDIA H100 GPU. Output logits were then transformed into a probability distribution by applying the softmax function over the entire matrix, instead of at each position—a key design decision and notable departure from standard language modeling that achieved strong empirical performance by preserving each position’s propensity to mutate (Methods).

### Evaluation metrics

We introduce two evaluation metrics to assess the extent to which antiGen and other models correctly predict future viral mutations: *next-mutation recall* and *position-specific scoring matrix (PSSM) precision*. Next-mutation recall measures how well a model can predict the next single amino acid substitution(s) relative to a given starting sequence. It is defined as the proportion of mutations across all testing examples that lie in the top q-quantile of predicted mutations, for 0≤q≤1 (Figure 2A; Equation 2). Since next-mutation recall is a function of q, a single summary statistic may be obtained by calculating the area under the recall curve (AUC), with a perfect predictor attaining an AUC of 1 and a uniformly random model scoring 0.5. The recall for individual values of q can be used for more detailed analyses, e.g., to assess which model’s top 10% of predicted mutations are most reliable.

To test longer-term evolutionary forecasting, we assessed each model’s ability to predict the set of mutations across all leaves on the testing tree that are descended, either directly or indirectly, from a specified ‘root’ sequence that emerged within the testing window (Figure 2F). To evaluate this metric, we first calculated the PSSM of sampled sequences descended from the root and collected prior to time t, as a function of t. Each PSSM is defined as a nonnegative integer matrix whose entry (i,j) denotes the number of times position i exhibited amino acid j across all sampled sequences satisfying the aforementioned ancestral and temporal criteria (Gribskov et al., 1987). We then scored a model’s ability to predict the PSSM using precision—specifically, the probability that a mutation in the top decile of predictions exceeds a given observation threshold M in the PSSM (Equation 3). The M=100 case matches the evaluation used in EVEscape (Thadani et al., 2023) on SARS-CoV-2 data, the only difference being that we evaluate all possible amino acid substitutions instead of restricting to those explainable by single nucleotide polymorphisms. Moving forward, we term this metric ‘PSSM precision’ to emphasize that the precision is computed over all direct and indirect descendants of the fixed root. As PSSM precision is a function of t, a single summary statistic may be obtained by calculating the maximum value it attains over all t, which is equivalent to the probability of a mutation in the top quantile of predictions emerging *eventually*. Again, this summary statistic is bounded above by one.

Finally, in accordance with standard evaluation of protein language models (Notin et al., 2023), we assessed the Pearson correlation between the model output and phenotypic measurements from a DMS, provided that a DMS was available for the query. We observed that for SARS-CoV-2 in particular, DMS experiments assigned relatively similar scores to all possible substitutions at a given position. Hence, to model conservation alone, we also measured the correlation after transforming each model’s output by replacing the (i,j)th entry with the average over all entries at position i. We tested both versions of correlation using the original as well as log-transformed mutation scores.

We applied these evaluation metrics to antiGen, existing protein language models (Lin et al., 2023; Hopf et al., 2017; Notin et al., 2022; Fraser et al., 2021; Rao et al., 2021; Thadani et al., 2023; Ito et al., 2025), DMSs (Starr et al., 2022; Yu et al., 2026; Simonich et al., 2026), and two bioinformatic baselines that do not require any model training or experimental data collection: a BLOSUM62 substitution matrix (Henikoff and Henikoff, 1992) and a PSSM of all training sequences. For SARS-CoV-2, we calculated all evaluation metrics based only on mutations to the receptor-binding domain (RBD) of the spike protein due to its high virological and clinical relevance, and to compare to previous models (Thadani et al., 2023); for all other viruses, we analyzed full protein sequences. For mathematical details of the evaluation metrics and further information on the bioinformatic baselines, see Methods.

### SARS-CoV-2 mutation forecasting

Given the impact of SARS-CoV-2 evolution on public health planning, we first applied antiGen to SARS-CoV-2 mutation forecasting. antiGen achieved state-of-the-art performance on numerous variant prediction tasks as compared to current computational, experimental, and bioinformatic methods, based on training data from the first year of the pandemic (323,950 sequences) and testing data from all subsequent years (9,011,678 sequences). According to next-mutation recall (Figure 2B and 2D), antiGen outperformed all existing approaches by AUC, though we note that a PSSM constructed from the training data showed the most reliable top decile of predictions. In addition to overall next-mutation recall, we also computed the same statistic for *de novo* mutations, i.e., mutations never before seen in the training data. antiGen once again outperformed all other methods in this case (Figure 2C and 2E). Under several other variations of this evaluation, such as masking of stop and gap codons (which are not part of the vocabulary of some existing methods), antiGen maintained leading performance (see Figure S1 and S3).

We next measured antiGen’s ability to forecast the mutations observed in population-wide genomic surveillance, a prediction task with clear epidemiological utility that—unlike next-mutation recall—is independent of the inferred evolutionary history of the testing data. In terms of PSSM precision, the metric we designed for this evaluation, antiGen once again outperformed existing methods. We computed PSSM precision with the root genotype set to the BA.2 (Omicron) wild-type, a variant of concern that emerged in late 2021 and is ancestral to nearly all SARS-CoV-2 cases today (Hadfield et al., 2018). As before, we segmented our analysis into prediction of all mutations versus *de novo* mutations only. For predicting any mutation to appear at population frequency ≥ 1 downstream of BA.2 (Figure 2G and 2I), antiGen performed state-of-the-art, marginally surpassing the PSSM in terms of its maximum value. For *de novo* mutations, which by definition cannot be predicted better than random by the PSSM, antiGen surpassed all other models substantially (Figure 2H and 2J). To assess the emergence of more prevalent mutations, we also conducted the same two evaluations with a population frequency cutoff of 100 genomes (Figure S2–S3); in this case, the PSSM and EVE marginally outperformed antiGen. However, fine-tuning EVE on the antiGen data and objective (as discussed in the following section) further improved performance, indicating a hybrid approach may be best for certain tasks.

We finally assessed antiGen’s ability to predict mutation effects as measured *in vitro* by calculating correlation with DMS experiments. Among computational and bioinformatic methods, antiGen evaluated on the BA.2 wild-type achieved the highest correlation with a DMS for the BA.2 variant, both for expression and binding data (Figure 2K and S4). For antiGen (and several other models), log-transforming the inferred probabilities and then setting each entry of the resulting matrix to the average value at its position on the protein sequence achieved the highest correlation, suggesting that strong performance under this evaluation derives from learning conservation. No computational or bioinformatic method was a better predictor of a BA.2 DMS than a BA.1 DMS (Figure S4), though we note that both of these DMS studies were conducted by the same group in similar experimental systems.

### Improving performance with fine-tuning and gating

Across our SARS-CoV-2 results, no single model achieved the highest performance across all evaluations, even though antiGen’s performance was consistently strong. We next tested methods of combining the strengths of multiple models for superior overall predictive power. One way to do so is fine-tuning—that is, using an existing pre-trained model and continuing its training with both the antiGen loss function and data. Applying this technique to EVE, Tranception, and ESM-2 (Methods), we observed that antigen-fine-tuned EVE performed state-of-the-art on three evaluations: (i) PSSM precision for *de novo* mutations downstream of BA.2 with frequency cutoff 100 (Figure 2L), (ii) next-mutation recall when single-amino-acid indels and nonsense mutations are not considered (Figure S3C), and (iii) BA.2 DMS correlation for both binding and expression, excluding other DMSs as predictors (Figure S4).

We moreover observed that while antiGen generally achieved the highest next-mutation recall AUC, the PSSM—despite being agnostic to the query sequence—exhibited superior recall based only on the top decile of predictions (Figure 2B). Experimenting with train-test cutoffs other than one year into the pandemic, we noticed that this performance discrepancy widened as the training data volume decreased (Figure 2M). We reasoned that antiGen and PSSM-based prediction have complementary strengths, potentially enabling a hybrid model that performs well in both low- and high-data settings.

We therefore designed a *gated* predictor that blends the per-position outputs of two models through a learned, position-specific weight (Methods). Based on a train-test cutoff one year into the pandemic, the gated model surpassed the PSSM on next-mutation recall (Figure 2M). Moreover, sweeping the train-test cutoff throughout the first six years of the pandemic, the gated model consistently matched or exceeded the performance of either antiGen or the PSSM, confirming its robustness to different levels of training data. This result also held for next-mutation recall based on the top 20% and 50% of the most likely mutations to emerge (Figure S5B–C) and for precision in predicting mutations that emerge at least once (Figure S6A–D). For predicting mutations that appeared at least 100 times, the gated model performed similarly to the PSSM alone (Figure S6E–H). The same trend in performance held for antiGen with a protein language model partner: gating antiGen with a partially fine-tuned 650M-parameter ESM-2 outperformed either component alone across all train-test splits based on next-mutation recall at the 10% quantile threshold (Figure S5A), though did not surpass the PSSM until over a year into the pandemic. This suggests that the PSSM supplies most of the complementary signal to antiGen in the early pandemic, enabling the gated predictor to have robust performance in both data-limited and data-rich regimes.

### antiGen improves as surveillance data increases

Because antiGen’s performance can improve with additional data, we further assessed the level of genomic surveillance necessary for antiGen and its fine-tuned or gated versions to deliver reliable performance over baselines. The earliest train-test split date we tested, four months into the pandemic (April 27, 2020), yielded a training tree with 56,299 sequences containing 2,228 total mutations. At this split, we found that antiGen alone already outperformed EVEscape and the BLOSUM62 and PSSM baselines in terms of PSSM precision with observed population frequency ≥ 1, as well as next-mutation recall based on the top 20% and 50% quantiles (Figure S5B–C and S6A). antiGen with the PSSM gate performed even better than either of its two components based on these evaluations. For next-mutation recall based on the top decile, however, the antiGen/PSSM gate and the PSSM alone performed similarly, followed by the BLOSUM62 matrix, antiGen alone, and finally EVEscape (Figure 2M and S5A). It was not until the one year and four month cutoff (1,067,026 sequences, 6,234 total mutations) that we observed the antiGen/PSSM gate consistently overtake the PSSM based on top-decile recall.

We note that antiGen’s training examples depend on the set of inferred mutations to occur within the training window, not the number of raw sequences. Our findings indicate that uncovering only a few thousand mutations through genomic surveillance (corresponding to the first few months of the COVID-19 pandemic, in this case) is sufficient for training antiGen to state-of-the-art performance on SARS-CoV-2. However, leading performance on a select subset of evaluation metrics may benefit from greater volumes of data and, more generally, antiGen’s predictions improve as more data is collected. For a complete summary of model performance by training data statistics, see Figure S5.

### Experimental validation of predicted SARS-CoV-2 variants

Given that antiGen can predict future mutations relative to the present strain, we sought to test novel mutations introduced on top of a currently circulating genetic background to further assess the model’s ability to generalize to unseen evolutionary sequence space. We designed an experimental workflow to test whether antiGen-predicted spike variants could retain pseudovirus infectivity (Figure 3A; Methods) (Dadonaite et al., 2023). We queried antiGen using the sequence of XFG, a major circulating SARS-CoV-2 variant at the time of this study (World Health Organization, 2025), and tested predicted mutations within the RBD of the spike protein (Figure 3B).

Because infectivity measured from equal volumes of pseudovirus supernatant can be confounded by differences in particle abundance (Youssef et al., 2025), we quantified each pseudovirus stock by flow virometry (Arakelyan et al., 2013; Maltseva and Langlois, 2022) and compared variants at matched physical particle inputs within a linear operating window shared across the panel of variant pseudoviruses (Figures S7, S8, S9, S10, S11; Methods). Here, “particle” refers to a pseudovirus-sized nanoparticle enumerated by side scatter (SSC)-based flow virometry, and “infectivity” refers to luciferase reporter signal (relative luminescence units; RLU) normalized to such physical particle input, which serves as a readout of pseudovirus entry (Figure 3A).

Using this workflow, we tested the infectivity of the ten highest-ranked antiGen-predicted mutations across all candidate RBD mutations, together with the ten highest-ranked *de novo* mutations, defined as mutations not observed in the training data from the first five years of the SARS-CoV-2 pandemic (Figure 3B). The majority of antiGen-predicted XFG spike variants retained measurable pseudovirus infectivity, with only two *de novo* variants exhibiting severe functional defects, indicating that antiGen predominantly proposes mutations that remain compatible with spike-mediated entry rather than broadly deleterious sequence changes (Figure 3C). These results were generally consistent with our PSSM precision estimates for antiGen, which reached approximately 90% for all predicted mutations and 70% for *de novo* mutations (Figure 2G–H).

Individual mutations produced a broad spectrum of phenotypes, from severe loss of infectivity to levels exceeding the XFG wild type. Compared with the distribution of random mutations in a DMS reference dataset (Methods), antiGen predictions were significantly enriched for tolerated mutations: six of the top ten overall predictions showed infectivity comparable to or greater than the XFG wild type ($p<6.9\times 10^{-4}$), as did four of the ten top-ranked *de novo* predictions (p<0.019). The *de novo* predictions showed greater variability, including one variant with more than a two-fold increase in infectivity and two with no measured infectivity.

While our *in vitro* system does not assess every determinant of epidemiological fitness, including replication capacity, pathogenicity of live virus, or mutational effects on antigenicity that contribute to antibody escape (Carabelli et al., 2023), these findings provide experimental support for the functional plausibility of antiGen-predicted spike variants and further validate the model’s ability to propose viable mutations in previously unobserved evolutionary sequence space.

### Mutation forecasting for endemic viruses

With statistical and experimental confirmation of antiGen’s ability to forecast SARS-CoV-2 evolution, we next applied the model to predict evolutionary changes to three other common endemic viruses that likewise pose a challenge to public health: H3N2 seasonal influenza, RSV (subgroup A), and dengue virus (serotype 2). For the H3N2 hemagglutinin data with a train-test split in 2022 (61,446 training sequences, 140,600 testing sequences), antiGen achieved higher next-mutation recall in all quantiles than all baseline methods: the training data PSSM, PSSM-based conservation scores per position, a BLOSUM62 matrix, EVE (with MSA consisting of all sequences in the training window), and a DMS experiment from a 2022 strain (Yu et al., 2026) (Figure 4A). While improvement over the PSSM was modest based on all (i.e., previously seen and unseen) mutations, after filtering only to *de novo* mutations, antiGen outperformed the other methods far more substantially (Figure 4D). To calculate PSSM precision, we chose the J subclade as our variant of interest because it emerged within the testing window and is ancestral to most sequences today (Hadfield et al., 2018). Using an observed frequency threshold of 1 sequence, antiGen again outperformed the PSSM and DMS, with marginal improvement across all mutations (Figure 4G) and major improvement for *de novo* mutations alone (Figure 4J). We repeated the next-mutation recall analysis with gaps and stop codon predictions masked (Figure S12G,J) and the PSSM precision analysis with observed mutation frequency ≥ 10 (Figure S12S,V); antiGen retained state-of-the-art performance.

Despite substantially less available data for RSV fusion protein and dengue virus envelope protein, antiGen also maintained strong predictive performance relative to the baselines across almost all evaluation metrics. For both viruses, we selected variants of epidemiological significance (for PSSM precision calculation) that had substantial numbers of representative or descendant sequences, yet originated recently enough to allow for a sufficient training window prior to emergence. The A.D.1.6 RSV clade (Zhuang et al., 2025) with a 2020 cutoff (3,913 training sequences, 14,867 testing) and the 2II_F.1.1.2 dengue minor lineage (González-Elizondo et al., 2025; Grubaugh et al., 2024) with a 2019 cutoff (11,898 training, 14,505 testing) satisfied these criteria. For both RSV and dengue, antiGen achieved higher overall next-mutation recall than the PSSM methods, the BLOSUM62 matrix, EVE, and, in the case of RSV, an available DMS (Simonich et al., 2026) (Figure 4B–C). Similarly, the improvement in antiGen recall curves over the PSSM was substantially more pronounced after filtering to *de novo* mutations (Figure 4E,F), though AUCs for RSV fusion and dengue virus envelope proteins were moderately lower than the equivalent evaluations for influenza. In terms of PSSM precision with population frequency 1 (Figure 4H,I), antiGen outperformed the PSSM for dengue throughout the whole testing window but slightly underperformed the PSSM for RSV between 2020 and 2024. Again, filtering to *de novo* mutations revealed a greater improvement in antiGen over existing methods (Figure 4K,L), though we note that the actual proportions of *de novo* mutations in the top decile of predictions to be observed by 2026 were modest for dengue (about 6%) due to limited overall genetic diversity in the testing data. For further variations on these evaluations, see Figure S12. In total, these results suggest that antiGen is a strong predictive method for viruses across a broad range of evolutionary profiles.

## Discussion

antiGen is a highly accurate method for forecasting future mutations to viral proteins, capable of leveraging all presently available genomic surveillance data and improving as new sequences are collected. antiGen outperformed DMS experiments, other machine learning models, and bioinformatic approaches for predicting both the next mutations to alter a protein sequence and mutations further downstream, based on pandemic and endemic viral datasets. In some cases, combining antiGen with existing methods via fine-tuning or gating further improved performance. Experimental validation confirmed that most antiGen-predicted SARS-CoV-2 spike variants remained compatible with pseudovirus infectivity.

From a model design perspective, antiGen showcases the utility of training directly on phylogenetic trees as opposed to MSAs or unaligned sequence databases. Our training data format—inferred mutations relative to their parent sequence, according to the phylogeny—remains robust to different levels of genomic surveillance for different variants. While the training data generation pipeline was designed for viral proteins, it could be applied to any set of sequences with high pairwise identity, opening a wide range of possible applications including evolutionary forecasting for bacterial pathogens and cancer. Our method assumes the training sequences are closely related evolutionarily to the query sequences whose future evolutionary trajectories we wish to predict, which may limit its applicability to, for example, novel pathogens with scarce surveillance data.

While antiGen provides reliable predictions for mutations that appear in the future or maintain infectivity *in vitro*, we emphasize that single-substitution variants are unlikely to pose unprecedented biosecurity risk as outlined by recent United States government proceedings (National Science and Technology Council, 2024; Exec. Order No. 14,292, 2025). Single amino acid substitutions leave sequence homology high enough that existing DNA synthesis screens that incorporate homology-based threat detection methods (such as BLAST search (Altschul et al., 1990)) will flag them with comparable accuracy to existing strains (Gretton et al., 2025). Mutation prediction is moreover an established field, and has limited applicability to designing more transmissible or lethal pathogens as most non-deleterious point mutations have only a small impact on fitness (Sanjuán et al., 2004). Nonetheless, we acknowledge that biosafety and biosecurity defenses can be imperfect and support efforts to develop responsible biological artificial intelligence (Responsible AI for Protein Design Community, 2023) without restricting important public health research.

Through future studies, we aim to better understand the mechanism by which antiGen predicts future mutations. Given the comparable performance of antiGen and the PSSM under certain evaluations, we hypothesize that some component of the antiGen architecture simply learns whether a given mutation at a given position has been seen in training. However, the fact that antiGen could still accurately forecast *de novo* mutations means it must also learn other evolutionary constraints, possibly reflecting conservation, chemical similarity of amino acids, distance in the genetic code, or other yet unknown factors we hope to determine. Moreover, as antiGen has a low number of trainable parameters in the context of machine learning for biology, further experimentation with larger, more sophisticated architectures may help explicitly capture these features.

Another area of future development lies in the phylogenetic tree inference method underlying antiGen’s training data. In this study, we used a parsimony-based algorithm to construct large-scale trees, from which we extracted evolutionary changes. While this is an established heuristic, a more sophisticated and potentially more robust approach would involve joint inference of the tree together with the underlying sequence variation and fitness model. With an epidemiological prior on the tree (Stadler et al., 2013) that takes into account transmission rates that vary by genotype, this joint inference procedure could simultaneously serve the purpose of reconstructing the evolutionary past, identifying concerning mutations in sequences at present, and forecasting variants liable to sweep the population in the future.

From a practical perspective, antiGen could be used to develop more effective interventions for viral diseases by, for example, anticipating mutations that escape from antibody therapeutics or by selecting vaccine strains that contain present and future mutations. Emerging methods for *de novo* antibody design (Mille-Fragoso et al., 2026) could be combined with antiGen predictions to proactively generate therapeutic antibodies against both historical and future variants of a viral protein.

antiGen’s improved performance with additional training data underscores the importance of widespread genomic surveillance data being available to the broader scientific community for key public health modeling and decision-making. Considering its utility for charting the near-term and longer-term evolutionary trajectory of viral pathogens, antiGen could better equip epidemiological authorities to preempt future variants that pose a serious risk to human health.

## Methods

### Phylogenetic tree construction and training data generation

As inputs, antiGen accepts (a) a “guide” phylogenetic tree with times and inferred sequences assigned to every node and (b) an MSA with a sample collection date for each genome. For the four viruses studied in this paper (SARS-CoV-2, H3N2 influenza, RSV A, and dengue virus 2) we obtained the guide trees from Nextstrain (Hadfield et al., 2018) (dataset IDs ncov/open/global/6m, seasonal-flu/h3n2/ha/12y, rsv/a/genome/all-time, and dengue/denv2/genome, respectively). The Auspice format JSON files for phylogenetic trees available on Nextstrain contain the inferred times of every node and the inferred evolutionary changes along each branch, from which the sequence at each internal node may be reconstructed. The MSA for SARS-CoV-2 was compiled from all available public genomes and downloaded from LAPIS (Chen et al., 2023). MSAs for the other three pathogens were obtained by downloading all complete sequences not subject to data embargo from GISAID (Shu and McCauley, 2017; Wallau et al., 2023) and aligning them with Nextclade (Aksamentov et al., 2021).

To produce pandemic-scale phylogenetic trees efficiently, we adapted a compact data structure known as a “mutation tree” (Jahn et al., 2016) or “perfect phylogeny” (Specht and Palacios, 2026; Gusfield, 2002) to record inferred mutations ancestral to the observed sequences. This structure consists of a directed tree, where each node represents an inferred past genotype, and each parent-to-child edge represents the evolution of the child genotype from its ancestor by way of one or more mutations—specifically, amino acid substitutions, for our purposes. We note that the terms “mutation tree” and “perfect phylogeny” have primarily been used in the context of the infinite sites model, in which mutations always affect different sites on the genome; here, we relax that assumption. A mutation tree may be obtained from a phylogenetic tree with reconstructed ancestral states at the internal nodes by collapsing all edges with zero mutations. In addition to the evolutionary changes at each node relative to its parent, we also record the collection date of each sampled sequence of each genotype on the mutation tree.

We obtain an approximate large-scale mutation tree by first extracting the mutation tree of the guide phylogeny, then iteratively attaching every sequence in the MSA in order from the earliest to latest sample collection date (Figure S13A). For the initialization phase, we construct the mutation tree of the guide phylogeny by simply collapsing all parent-child edges without inferred mutations. In the attachment phase, we compute the parsimonious attachment point of a sequence in the MSA by considering the four possible ways that the mutation tree can be augmented with a new sequence: (a) the new sequence matches the sequence at an existing node; (b) the new sequence should be attached as a new leaf whose parent is an existing node; (c) the new sequence should be attached as an intermediate of an existing parent-to-child edge; and (d) the new sequence should be attached as a new leaf whose parent is an intermediate of an existing parent-to-child edge. Note that in scenario (d), the sequence at the new internal node is selected to minimize the total number of mutations to its three adjacent nodes. In practice, we observed that the overwhelming majority of attachments fell into category (a) (about 96% for SARS-CoV-2 data), which does not alter the tree topology and is hence cheap—the main efficiency improvement over a binary tree as the underlying data structure. Additionally, we store the sequence at each node in the mutation tree explicitly (in contrast to, e.g., UShER (Turakhia et al., 2021), which stores changes relative to parent)—a design decision that sacrifices memory efficiency for idiomatic GPU parallelization. To compare our method’s performance to UShER (Figure S14), we also adapted our tool to accept the nucleotide rather than the amino acid alphabet, though the nucleotide version was not otherwise used in this study.

As the mutation tree is grown, we concurrently track and update inferred times at each node which are later used to define the train-test split. This is in contrast to UShER, which does not account for sample collection dates. The node time assignment is conducted in two steps: an ‘earliest-case assignment’ during the initialization and attachment phase, followed by a ‘latest-case update’ after tree augmentation completes. The purpose of the earliest-case assignment step is to detect sequences in the MSA whose reported collection date is implausibly early based on molecular dating. It is implemented as follows: first, in converting the guide tree to an initial mutation tree, whenever a parent-to-child edge is collapsed into a single node, that node inherits the time of the parent. Then, in the attachment phase, the time of a newly attached leaf is set to the maximum of its reported sample collection date and the time of its parent node. For attachment case (d), where two new nodes are created in the mutation tree, the new internal node (not the leaf) is assigned the same time as its parent. The rationale here is that the reported leaf time should be adjusted only if necessary to avoid a parent-child edge where the parent’s time exceeds the child’s time. This temporal update affected only about 2% of sequences in the SARS-CoV-2 dataset.

While the ‘earliest-case assignment’ logic corrects collection dates that are too early, setting the time of each internal node to the minimum possible emergence time of its representative sequence may cause unobserved ancestral genotypes that emerged after the train-test split threshold to appear erroneously in the training data. To fix this, the ‘latest-case update’ step then resets the time of each node to the minimum time of all leaves descended (directly or indirectly) from that node via post-order traversal. This correction avoids data leakage by pushing forward internal node times to as late as they can possibly be, yielding a worst-case scenario in terms of the sequences available for training.

Training and testing examples were generated by (a) separating testing subtrees from the training subtree by splitting all edges whose parent time lies in the training window and whose child time lies in the testing window, (b) randomizing the order of mutations for edges with two or more of them, and (c) storing each sequence together with the mutations inferred to have affected it across the training and testing mutation trees as an example (Figure S13B). Step (b) is essential for avoiding the case in which sequences collected after the train-test cutoff inform the ordering of the mutations along a branch that lies entirely within the training window, which would constitute data leakage. We note that converting the mutation trees back into representative phylogenetic trees would make no difference in terms of the examples generated; hence we did not implement this step. Finally, as an extra safeguard, we verified that no (position, amino acid) pairs that first emerged after the train-test cutoff according to surveillance data were erroneously reported to have altered genotypes within the training window.

For the gating analysis, we additionally generated training and testing example sets at a sweep of 18 evenly spaced train-test cutoff dates spanning 0.35 to 5.94 years after the SARS-CoV-2 most recent common ancestor (MRCA; December 23, 2019), holding all other data-generation settings fixed, in order to characterize model performance as a function of the portion of the evolutionary history available at training time.

The training data generation pipeline was written in C++17 and CUDA (version 12.6) with the nlohmann /json package (Lohmann, 2025).

### Model setup and loss function

Let L denote the length of the amino acid sequence of the protein of interest and let A denote the number of tokens in the amino acid alphabet (A=22 for our purposes: the 20 standard amino acids, the stop codon, and a gap token). Adopting statistical notation, we define antiGen to be a function $F_{\theta }\;:\{1,\ldots ,A{\}}^{L}\to {\left[0,\;1\right]}^{L\times A}$ indexed by unknown, learnable, high-dimensional real parameters θ. The function $F_{\theta }$ takes in an amino acid sequence, represented as a vector of L tokens, and returns an L×A matrix of probabilities specifying the joint distribution over the position at which an amino acid substitution occurs and the replacement token for the amino acid at said position. We write the probability under the model of replacing the token at position i∈{1,…,L} with token j∈{1,…,A} in a sequence $q\in {\left\{1,\ldots ,A\right\}}^{L}$ as $F_{\theta }{\left(q\right)}_{ij}$. By convention, we assume that entries of the matrix $F_{\theta }(q)$ that do not represent actual mutations (i.e., (i,j) for which $j=q_{i}$, corresponding to degenerate substitutions where the mutant amino acid is the same as the original amino acid) are set to zero.

We defined the loss to be the negative log likelihood (NLL) of the mutations in the training data given θ, treating each mutation as an independent observation, given the phylogeny. Formally, let ℰ denote the set of training examples, where an example e∈ℰ consists of a query sequence $q^{\left(e\right)}\in {\left\{1,\ldots ,A\right\}}^{L}$ and a matrix $M^{\left(e\right)}\in Z_{\geq 0}^{L\times A}$ whose (i,j) th entry $m_{ij}^{\left(e\right)}$ is the number of times a mutation at position i to amino acid j was observed in training example e. As shorthand, for e∈ℰ, let $F_{\theta }(e)\in {\left[0,\;1\right]}^{L\times A}$ denote the evaluation of $F_{\theta }$ on the sequence $q^{\left(e\right)}$. The loss is then given by

(1) $$loss(\theta )\;:=-\frac{1}{Z}\sum_{e\in ℰ}\sum_{i=1}^{L}\sum_{j=1}^{A}m_{ij}^{\left(e\right)}log\left(F_{\theta }{\left(e\right)}_{ij}\right)$$

where Z is the total number of mutations across all training data, given by

$$Z:=\sum_{e\in ℰ}\sum_{i=1}^{L}\sum_{j=1}^{A}m_{ij}^{\left(e\right)}.$$

We minimized this loss function via stochastic gradient descent in order to yield an approximate maximum likelihood estimate $\overset{ˆ}{\theta }$ of θ.

### Training and fine-tuning

Here, we supply the technical details for training antiGen from scratch and fine-tuning existing models on the antiGen objective (or simply evaluating them as-is). We provide a brief description of each model as well.

- **antiGen** (Transformer trained from randomly initialized parameters). A small pre-norm encoder-only Transformer (token + absolute positional embeddings, $d_{\text{model}}=128$, 2 layers, 4 heads, FFN dim 512, dropout 0*.*1) with a linear head onto the 22-symbol amino-acid vocabulary. Because it has no pre-training stage, every parameter was initialized at random and trained end-to-end with the loss function of Equation 1 for 32 epochs at learning rate 10^−4^, batch size 64.
- **ESM-2.** A masked protein language model pre-trained on UniRef50 (Lin et al., 2023). We used the 35M-parameter (facebook/esm2_t12_35M_UR50D) and 650M-parameter (facebook/esm2_t33_650M_UR50D) checkpoints. We projected the per-position hidden states onto the amino-acid vocabulary using ESM-2’s pre-trained masked-language-model head, with its output logits restricted and reordered to our 22-token alphabet. We evaluated four configurations:
  - *No fine-tuning*: the pre-trained backbone and pre-trained head are evaluated zero-shot, with no gradient updates.
  - *Frozen* (epochs 32, lr 10^−3^): backbone gradients were disabled; only the linear head was updated.
  - *Partial* (epochs 32, lr 10^−4^): the last 4 Transformer blocks and the head were unfrozen; earlier layers remained fixed.
  - *Full* (epochs 32, lr 10^−5^): every parameter was trainable.

Batch sizes were 32/32/32/16 for the 35M model and 4/4/4/4 for the 650M model, respectively.

- **EVE.** A deep variational autoencoder (VAE) over an MSA, originally proposed for human variant-effect prediction (Fraser et al., 2021). For each dataset we re-fit a fresh EVE VAE from scratch (encoder hidden sizes [2000*,* 1000*,* 300], latent dimension 50, decoder [300*,* 1000*,* 2000] with Bayesian linear layers, a depth-40 Conv1d output, and a learned temperature) on the dataset’s MSA restricted to sequences collected within the training window, i.e., the sequences at the leaves of the training tree. Sequences were reweighted with EVE’s θ=0.01 identity-clustering scheme. The VAE was pre-trained for 4 × 10^5^ steps at batch size 256 under the EVE ELBO (reconstruction NLL + latent KL + Bayesian-decoder $KL/N_{\text{eff}}$) with Adam at learning rate 10^−4^. For model evaluation, we used these pre-trained decoder log-probabilities directly. For fine-tuning, we then unfroze the VAE and continued training the decoder log-probabilities against our mutation-emergence loss for 32 epochs at learning rate 10^−4^, batch size 32.
- **Tranception.** A causal autoregressive protein language model with grouped ALiBi positional encoding and Inception-style depthwise convolutions in attention ($d_{\text{embd}}=256$, 8 heads, 6 layers, dropout 0*.*1) (Notin et al., 2022). As with EVE, we pre-trained the Tranception architecture on each dataset’s date-filtered MSA for 1*.*5 × 10^5^ steps with AdamW (lr 3 × 10^−4^, weight decay 10^−4^, linear warmup followed by cosine-like decay, batch size 32); per-position log-likelihoods were averaged over the forward and backward scoring directions. For model evaluation, we used these scores directly. For fine-tuning, we continued training the same parameters against our mutation-emergence loss for 32 epochs at learning rate 10^−4^, batch size 32.
- **EVcouplings.** A mean-field direct coupling analysis model fit by pseudo-likelihood maximization on the same date-filtered MSA (capped at 10^4^ sequences) via the plmc binary (Hopf et al., 2017). Each candidate substitution was scored by its delta-Hamiltonian ΔH=Δh+ΔJ, combining single-site fields and pairwise couplings. We did not fine-tune EVcouplings.
- **CoVFit.** An ESM-2 backbone fine-tuned on SARS-CoV-2 spike deep-mutational-scanning data (Ito et al., 2025), used here as a fixed predictor that cannot adapt to different query sequences (as it was not designed to do so). We extracted the BA.2 testing-root spike sequence, enumerated all single-amino-acid mutants over the 22-token alphabet, and invoked the published covfit_cli binary (fold 0, batch 16) to score the wild-type plus every mutant. Per-substitution scores were converted to a [sequence length] × [alphabet size] matrix of changes in fitness, i.e., fitness(mutant) − fitness(wild type). Positions beyond CoVFit’s 1022-residue context window were marked with a sentinel and excluded from evaluation. CoVFit is SARS-CoV-2 specific and is therefore reported only on the SARS-CoV-2 datasets; no fine-tuning was performed.

All trainable models above were optimized with Adam (Kingma and Ba, 2015) (default PyTorch hyperparameters: β=(0.9,0.999), $\varepsilon =10^{-8}$, no weight decay), gradient norm clipped to 1*.*0, no learning-rate schedule, and no mixed precision unless otherwise specified. Data loaders used 4 worker processes, pinned memory, and shuffled batches. We note that for antiGen as well as for many of the fine-tunes, although loss continued to exhibit a negative slope at the 32-epoch mark, training beyond this point offered little to no improvement in terms of our evaluation metrics, and sometimes damaged performance due to overfitting. Hence, for all models, we chose to train for a fixed 32 epochs. See Figure S15 for loss curves.

The training and evaluation pipeline was written in Python (version 3.12.2) with the following libraries: numpy (Harris et al., 2020), scipy (Virtanen et al., 2020), pandas (McKinney, 2010), matplotlib (Hunter, 2007), biopython (Cock et al., 2009), PyTorch (Paszke et al., 2019), and Hugging Face (Wolf et al., 2020).

### Evaluation metrics

Here, we provide mathematical details of the evaluation metrics used to assess antiGen and other models. We continue with the notation established in Methods, *Model setup and loss function*. We defined next-mutation recall to be the proportion of mutations across all testing examples that lie in the top q-quantile of predicted mutations, for 0≤q≤1. That is,

(2) $$recall(q)\;:=\frac{1}{Z^{\prime }}\sum_{e\in {ℰ}^{\prime }}\sum_{i=1}^{L}\sum_{j=1}^{A}m_{ij}^{\left(e\right)}1(F_{\overset{ˆ}{\theta }}(e{)}_{ij}\geq \zeta_{1-q}(F_{\overset{ˆ}{\theta }}\left(e\right))),\;q\in [0,\;1],$$

where ${ℰ}^{\prime }$ denotes the set of *testing* examples, $Z^{\prime }$ is the total number of mutations across all testing examples, i.e.

$$Z^{\prime }\;:=\sum_{e\in {ℰ}^{\prime }}\sum_{i=1}^{L}\sum_{j=1}^{A}m_{ij}^{\left(e\right)},$$

and $\zeta_{1-q}\left(F_{\overset{ˆ}{\theta }}(e)\right)$ denotes the (1-q)-quantile of the values in the matrix $F_{\overset{ˆ}{\theta }}(e)$ (with linear interpolation in the presence of ties).

For PSSM precision, we first give an exact definition of the PSSM. Here, the PSSM is computed for a specific time window within the testing dataset, but the same definition applies for the PSSM in the training window that is used as a predictor. The testing-data PSSM for all sequences descended from a given node and collected before a given time t is a matrix of dimension L×A with non-negative integer values, given by

$$PSSM(\text{node},t{)}_{ij}\;:=\sum_{s\in {𝒮}_{t}^{\prime }}s_{ij},$$

where ${𝒮}_{t}^{\prime }$ is the set of all sequences in the testing data collected before time t, and for $s\in {𝒮}_{t}^{\prime },\;s_{ij}$ is the indicator of sequence s having amino acid j at position i. PSSM precision, i.e., the proportion of predictions in the top decile observed at least M times in the testing window by time t, is then given by

(3) $$\text{PSSM}\;\text{precision}(\text{node},t,M):=\frac{\sum_{i=1}^{L}\sum_{j=1}^{A}1(\text{PSSM}(\text{node},t{)}_{ij}\;\geq \;M)1(F_{\overset{ˆ}{\theta }}(\text{node}{)}_{ij}\;\geq \;\zeta_{0.9}(F_{\overset{ˆ}{\theta }}(\text{node})))}{\sum_{i=1}^{L}\sum_{j=1}^{A}1(F_{\overset{ˆ}{\theta }}(\text{node}{)}_{ij}\;\geq \;\zeta_{0.9}(F_{\overset{ˆ}{\theta }}(\text{node})))}.$$

Here the notation $F_{\overset{ˆ}{\theta }}(node)$ denotes the evaluation of $F_{\overset{ˆ}{\theta }}$ on whatever testing example contains the node, as testing examples partition the testing tree. In this paper, the node argument is always chosen to be the earliest testing example whose sequence is classified as a given variant of interest.

To calculate these evaluation metrics on antiGen and other machine learning models, substitution probabilities were obtained as described in Methods, *Training and fine-tuning*, then passed into the above metrics. Note that one model, CoVFit (Ito et al., 2025), is only relevant to SARS-CoV-2 data with a traintest split of November 2023 and was hence omitted from Figure 2; for a comparison to antiGen with the same cutoff date, see Figure S16. Changes in binding and expression were used directly to compute evaluation metrics for DMS predictors, with each missing entry (i.e., a mutation to a given amino acid at a given residue for which no experimental measurement was available) masked in the quantile calculations. For the BLOSUM62 baseline, we constructed an L×A matrix of scores whose (i,j)th entry was the BLOSUM62 score of mutating the wild-type amino acid at position i to amino acid j. Note that this predictor cannot discriminate between positions or adjust its predictions based on the starting sequence. For the PSSM predictor, we constructed a PSSM based on all samples collected prior to the train-test cutoff date using the formula provided earlier in this section, with “node” being the global root of the training tree, t being the cutoff date, and ${𝒮}^{\prime }$ now representing the set of sequences collected *before* time t. The training data PSSM can be used to score the relative likelihoods of all possible mutations across all positions, but assigns a probability of zero to any mutation never seen in training data and, once again, is agnostic to the starting sequence. Hence, we also modeled conservation alone by averaging the mutation counts for each position, an approach that sacrifices discrimination between different amino acid identities for the ability to predict mutations that were not observed in the training data. The DMS, BLOSUM62, and PSSM prediction matrices can be normalized to sum to 1 so that they indeed represent a probability distribution over substitutions; in practice, this was never needed because all of our evaluations depend only on the ranks of the substitutions or unnormalized substitution probabilities.

### Gating antiGen with other models

To describe our gating scheme, we first establish some notation. Let $S^{\left(a\right)},S^{\left(b\right)}\in R^{L\times A}$ denote the presoftmax score (logit) matrices of two component models a,b evaluated on a query sequence (for a PSSM component, the (i,j)th entry of this matrix is $log\left({count}_{ij}+1\right)$), and let Z(⋅) standardize a matrix to zero mean and unit variance over its L×A entries, placing the two components on a common scale so the gate weight is interpretable as a genuine mixture. The gated score matrix S with entries $s_{ij}$ is defined as

$$s_{ij}=w_{i}Z{\left(S^{\left(a\right)}\right)}_{ij}+\left(1-w_{i}\right)Z{\left(S^{\left(b\right)}\right)}_{ij},$$

where $w_{i}\in [0,\;1]$ is a per-position gate, and the final probability matrix is obtained by applying the matrix-wide softmax to S just as for antiGen and every other model. We considered two parameterizations of the gate: The *static* gate learns one logit per position, $w_{i}=\sigma \left(\gamma_{i}\right)$ with each $\gamma_{i}\in R$ a free, trainable parameter and σ the sigmoid function,

$$\sigma (x)\;:=\frac{1}{1\;+\;exp(-x)},\;x\in R.$$

We initialized $\gamma_{i}=0$ (an equal blend of model components prior to any training); it is data-blind, adapting only to which component predicts better on average at each position.

The *uncertainty* gate is data-conditioned: at each position i it computes the Shannon entropies $H_{i}^{\left(a\right)},\;H_{i}^{\left(b\right)}$ of the two components' substitution distributions, where a lower entropy indicates a sharper, more confident prediction. It then maps the triplet $\left(H_{i}^{\left(a\right)},H_{i}^{\left(b\right)},H_{i}^{\left(a\right)}/(H_{i}^{\left(a\right)}+H_{i}^{\left(b\right)})\right)$ to $w_{i}=\sigma (\cdot )$ through a multilayer perceptron shared across all positions, allowing the model to defer to whichever component is locally more confident. The perceptron has a single hidden layer of width 16 with a ReLU nonlinearity (3 → 16 → 1); its output layer is initialized to zero, so that $w_{i}=0.5$ before training, as in the static gate.

For both gate variants, the gate parameters and any unfrozen model components were trained jointly under the same negative-log-likelihood objective and Adam optimizer used for the standalone models, while the PSSM (or any other bioinformatic baseline) was held fixed. We report three pairings for SARS-CoV-2 spike: antiGen and PSSM (16 epochs, batch size 64, capped at 10^4^ minibatches); and antiGen and both the 35M and 650M versions of ESM-2 (10 epochs, batch size 32, capped at 5 × 10^3^ minibatches). As the uncertainty gate did not yield any clear improvement in performance over the static gate, we report only the performance of the static gate.

### Cell culture

HEK293T/ACE2/TMPRSS2 cells were obtained from Peter S. Kim’s laboratory at Stanford University. Both HEK293T and HEK293T/ACE2/TMPRSS2 cells were grown in a 5% CO_2_ humidified incubator at 37°C in complete D10 medium consisting of Dulbecco’s Modified Eagle Medium (DMEM; Corning) supplemented with 10% fetal bovine serum (ThermoFisher), 2 mM L-glutamine, and 1% penicillin/streptomycin (Gibco).

### Lentiviral plasmids

An HDM-SARS2-Spike expression plasmid encoding the SARS-CoV-2 Wuhan-Hu-1 spike protein with a 21-amino-acid C-terminal truncation (Δ21) in the HDM expression backbone (“HDM-SARS2-Spike-delta21”; Addgene plasmid #155130) and the lentiviral packaging plasmids pHAGE-Luc2-IRES-ZsGreen (NR-52516), HDM-Hgpm2 (NR-52517), pRC-CMV-Rev1b (NR-52519), and HDM-Tat1b (NR-52518) were obtained from BEI Resources (NIAID, NIH; SARS-Related Coronavirus 2, Wuhan-Hu-1 Spike-Pseudotyped Lentiviral Kit V2, NR-53816). The Δ21 truncation enhances pseudovirus production and was applied uniformly across all spike variants (Crawford et al., 2020; Weidenbacher et al., 2022). Sequences of all plasmids used for pseudovirus production are provided in **Data S1**.

### Spike plasmid design and construction

The sequence of the Wuhan-Hu-1 spike protein within the HDM-SARS2-Spike-delta21 parental construct was replaced with the corresponding XFG spike sequence (GenBank accession YCJ32983.1; synthesized by Twist Bioscience) by PCR-based cloning. All antiGen-predicted mutations in XFG spike reported throughout this study are numbered relative to this wild-type XFG spike reference sequence, annotated accordingly on the plasmid map “HDM_SARS2_Spike_del21_XFG_antiGen” in **Data S1**. Individual amino acid substitutions were introduced into the XFG spike construct by site-directed mutagenesis using mutation-specific primer pairs, as described in **Data S1**. All primers were synthesized by Integrated DNA Technologies.

All cloning reactions were performed using NEBNext High-Fidelity Polymerase (New England Biolabs, Cat. No. M0544) for PCR amplification, followed by In-Fusion assembly (Takara Bio; #638948) or Gibson assembly (New England Biolabs; #E2621S) of fragments according to the manufacturers’ protocols. Recombinant plasmids were transformed into Stellar Competent Cells (Takara Bio; #636766) for propagation and plasmid production. Plasmid DNA was purified using either the QIAprep Spin Miniprep Kit (Qiagen) or the ZymoPURE II Plasmid Midiprep Kit (Zymo Research), according to the manufacturers’ instructions. The sequences of all constructs were confirmed by whole-plasmid Nanopore sequencing.

### Antibody production

Purified anti-SARS-CoV-2 RBD antibody BD55–1205 was obtained from GenScript. Purified anti-SARS-CoV-2 spike antibody CV10 was provided by the laboratory of Peter S. Kim, Stanford University. The heavy- and light-chain variable region amino acid sequences of all antibodies used in this study are provided in **Data S2**.

### Production of SARS-CoV-2 pseudovirus

SARS-CoV-2 pseudoviruses were generated using a five-plasmid lentiviral system in HEK293T cells. Cells were seeded at 6 × 10^6^ cells per 10-cm dish one day prior to transfection.

Plasmids were combined in D10 medium at the following amounts: 10 *μ*g pHAGE-Luc2-IRES-ZsGreen, 3.4 *μ*g spike expression plasmid, 2.2 *μ*g HDM-Hgpm2, 2.2 *μ*g HDM-Tat1b, and 2.2 *μ*g pRC-CMV-Rev1b. BioT transfection reagent (30 *μ*L; BioLand #B01–01) was added, and complexes were formed for 10 min at room temperature before addition to cells (Powell et al., 2026).

After 24 h, media was replaced with fresh D10 medium. Viral supernatants were harvested at 72 h post-transfection, centrifuged at 300×g for 5 min, and filtered through a 0.45 *μ*m PES filter. Viral stocks were aliquoted and stored at −80°C.

For concentration, filtered supernatants were processed using Lenti-X Concentrator (Takara Bio #631231) at a 3:1 ratio (1.8 mL supernatant + 0.6 mL reagent), incubated overnight at 4°C, and centrifuged at 1,500×g for 45 min. Pellets were resuspended in DMEM at one-tenth of the original volume, yielding approximately 10× concentrated stocks.

### Flow virometry

#### Flow virometry gating strategy

Pseudovirus-containing supernatants were diluted in 0.02 *μ*m-filtered Dulbecco’s Phosphate-Buffered Saline (DPBS; Gibco) prior to acquisition. Flow virometry was performed using a CytoFLEX Nano flow cytometer (Beckman Coulter) at a flow rate of 1 *μ*L/min for 30 s. Sample dilutions were adjusted as necessary to maintain the manufacturer’s recommended event rate. For all flow virometry analyses, events below the SSC-H threshold were excluded as instrument noise, after which singlet events were identified using SSC-W versus SSC-H gating (Figure S8A; Jungbauer-Groznica et al. (2026)). To establish a size-based pseudovirus gate, nanoViS low nanoscale sizing standards (Beckman Coulter #D03231) were first used to define bead-equivalent SSC boundaries (Figure S8B). Based on reported lentiviral particle diameters (80–100 nm; Transfiguracion et al. (2020)) and spike glycoprotein protrusion (about 20 nm; Tai et al. (2021)), the expected pseudovirus population was estimated to fall within an 80–140 nm size-equivalent range (Figure S8B).

To distinguish pseudovirus-associated events from background particles, three control preparations were analyzed: (i) A mock-transfected conditioned medium control consisting of culture supernatant collected from producer HEK293T cells without plasmid transfection was used to characterize extracellular vesicles and other cell-derived background particles; (ii) A ΔGag/Pol control, generated in the absence of the Gag/Pol packaging plasmids, served as a negative control for pseudovirus particle assembly; and (iii) A ΔSpike pseudovirus control, generated by omitting the spike-expression plasmid, served as a spike-negative pseudovirus reference.

For crude pseudovirus preparations, SSC distributions showed substantial overlap between wild-type (WT) and mock-transfected samples. However, following 10× concentration, the WT preparations exhibited a readily distinguishable particle population within the expected SSC range relative to the mock-transfected control, enabling confident definition of the SSC gate (Figure S8C). SSC-based particle gates were therefore determined using 10× concentrated pseudovirus preparations.

The final pseudovirus particle gate was defined on singlet events as SSC-H = 4 × 10^4^–4 × 10^5^, corresponding to the bead-calibrated size region enriched for WT pseudovirus events while excluding the majority of low-scatter events observed in mock-transfected conditioned medium. This SSC gate was used for all subsequent flow virometry analyses. The flow virometry results were analyzed using FlowJo v10.10.1 and v11.1.1 Software (BD Life Sciences).

#### Particle quantification and linearity assessment

Particle abundance was quantified using CytoFLEX Nano as the number of SSC-gated events acquired at a fixed flow rate of 1 *μ*L/min over a 30 s acquisition window, and converted to concentration based on the known acquisition volume, sample dilution factors, and sample concentration steps applied prior to acquisition.

To evaluate the quantitative operating range of CytoFLEX Nano particle enumeration, WT pseudovirus preparations were analyzed across a 1×–10× concentration series. Measured particle counts were compared with the expected proportional scaling, and linearity was assessed using linear regression. SSC-gated particle counts exhibited linear scaling across the tested concentration range (Figure S8D,E). Accordingly, 10× concentrated pseudovirus preparations were diluted 4-fold prior to acquisition, which maintained the event rate within the manufacturer’s recommended operating range while preserving measurements within the validated linear range.

#### Fluorescence-based validation of SSC-gated pseudovirus particles

To characterize spike-bearing particles within the SSC-defined nanoparticle population, 10× concentrated pseudovirus preparations were stained with monoclonal antibodies against the SARS-CoV-2 spike protein (Jungbauer-Groznica et al., 2026; Burnie et al., 2026). Because all antiGen-predicted mutations were located within the receptor-binding domain (RBD), the conserved S2-specific monoclonal antibody CV10 (human IgG1; Weidenbacher et al. (2022)) was used to detect spike-bearing particles while minimizing the potential effects of RBD mutations on antibody recognition. In parallel, the anti-RBD monoclonal antibody BD55–1205 (human IgG1; Jian et al. (2025)), a broadly neutralizing antibody recognizing a receptor-binding epitope distinct from all antiGen-predicted mutation sites analyzed in this study, was used to detect the display of intact RBD on pseudovirus preparations. 10-fold concentrated pseudovirus preparations were incubated with 1 *μ*g/mL CV10 or BD55–1205, as optimized by titration on WT or ΔSpike pseudovirus preparations (Figure S17A, B), followed by Alexa Fluor 647 (AF647)-conjugated goat anti-human IgG secondary antibody (Invitrogen #A-21445; 1:2,000 dilution). Following singlet gating, spike staining was analyzed using SSC-H versus AF647-H plots to assess the distribution of AF647-positive events across the SSC range (Figure S8A). AF647-positive and AF647-negative populations were identified using a fixed AF647-H fluorescence gate established from the ΔSpike pseudovirus, secondary antibody-only, and isotype control samples (Figure S17C). Stain performance was quantified using the stain index described in Maltseva and Langlois (2022) (Figure S17D):

$$\text{Stain}\;\text{index}=\frac{{MFI}_{\text{positive}\;\text{population}}\;-\;{MFI}_{\text{negative}\;\text{population}}}{{SD}_{\text{negative}\;\text{population}}}.$$

To verify single-particle detection, stained pseudovirus preparations were serially diluted prior to acquisition. Particle concentration decreased linearly with dilution, whereas the median fluorescence intensity (MFI) of the antibody-positive population remained constant, indicating that fluorescence measurements reflected individual particles rather than coincident events (Figure S17E).

Although antibody staining confirmed that the SSC-defined population was enriched for spike-bearing particles across all variants (Figure S18, Figure S19), fluorescence measurements were used to validate and characterize the particle population rather than for particle normalization. Antibody positivity reflects not only the presence of spike but also antibody accessibility, epitope recognition, and the amount and conformation of spike displayed on individual particles, all of which influence antibody binding. Consequently, antibody-based measurements may underestimate the number of infection-competent particles if functional spike-bearing particles exhibit reduced antibody-binding because of differences in epitope accessibility, antibody recognition, or spike presentation. Therefore, antibody-independent SSC-based particle counts (Figure S9) were used for particle normalization after establishing a common linear operating range across all variants.

### Particle-normalized infectivity assay

To compare infectivity across viral variants independently of differences in pseudovirus production, infectivity was measured as relative luminescence units per SSC-defined physical particle (RLU/particle).

A pilot infectivity study was performed using WT and all mutant pseudoviruses to define the linear operating range of the luciferase assay for each variant. Crude pseudovirus preparations were subjected to a 10-point, 3-fold serial dilution, and 100 *μL* of each dilution was added per well to HEK293T/ACE2/TMPRSS2 cells. Following infection, cells were incubated for 48 h at 37°C. Luciferase activity was then measured by adding 100*μ*L of BriteLite reagent (prepared as a 1:1 mixture with DPBS) to each well, and luminescence was quantified as relative luminescence units (RLU) using a Tecan Spark Multimode Microplate Reader.

The dilution-response relationship was evaluated to identify the range over which RLU scaled proportionally with viral input. Particle input per well was calculated from the flow virometry-measured particle concentration (Figure S9) and the corresponding inoculation volume for each dilution (Figure S10A). A construct-specific linear operating range was then determined for each pseudovirus variant. For the wild-type XFG pseudovirus and all variants except 475insC, the first four dilution points (1:1 to 1:27) exhibited an approximately linear relationship between particle input and RLU. In contrast, 475insC generated quantifiable luminescence only at the first two dilution points and was therefore excluded from defining the common linear operating range used for subsequent assay optimization (Figure S7).

A common particle concentration range was subsequently selected for a particle-normalized infectivity assay such that the second and third points of a four-point, 3-fold serial dilution series fell within this shared operating window. When the target starting particle concentration exceeded that of the unconcentrated pseudovirus supernatant, 10× concentrated pseudovirus prepared in DMEM was used to achieve the desired starting concentration. All samples had a final FBS concentration of 10% throughout the dilution series. Following infection, cells were incubated for 48 h at 37°C, and luciferase activity was measured as described in Figure S10B and Figure S11.

Infectivity was then calculated as:

$$\text{Infectivity}=\frac{\text{RLU}}{\text{particle}\;\text{input}}.$$

Because the second and third dilution points were predetermined to fall within the shared linear operating window across all constructs, these dilution points were designated *a priori* as the primary comparison range. Infectivity values from the two dilution points were averaged to generate a single infectivity estimate for each condition.

Relative infectivity was calculated by normalization to WT XFG measured in parallel within each experiment:

$$\text{Relative}\;\text{infectivity}=\frac{{\text{Infectivity}}_{\text{variant}}}{{\text{Infectivity}}_{\text{wildtype}}}.$$

Final values represent the mean across independent experiments.

### Statistical test for increased infectivity

As we did not measure the effects of arbitrary mutations on pseudovirus infectivity in our experimental pipeline, we defaulted to DMS data as a null distribution. To construct this distribution, we obtained DMS measurements of change in binding and expression for the two most recent available variants, KP.3 and LP.8 (Taylor and Starr, 2026). For each variant and each measurement type (i.e., binding and expression), we estimated the probability of a mutation increasing infectivity as the proportion of measured changes exceeding zero. For assessing the statistical significance of *de novo* mutations, we masked mutations already seen in the first five years of pandemic data prior to computing the proportion. For proportion x, the p-value for obtaining k out of n mutations that increase infectivity according to our experiments is given by

p-value=∑i=knnixi(1-x)n-i,

the binomial survival function evaluated at k. Per Figure 3, we have k=6,n=10 for the all-mutations case and k=4,n=10 in the *de novo* case. A summary of p-values is shown in Table S1. The p-value reported in the main text is the maximum of the p-values across variants and measurement type, rounded up to the nearest two significant figures.

### Ethics declaration and institutional review

Work with SARS-CoV-2 spike-pseudotyped lentiviral particles was approved by the Stanford University Institutional Biosafety Committee/Administrative Panel on Biosafety under APB protocol 6007-BH0126. The protocol covers production and use of lentiviral particles pseudotyped with SARS-CoV-2 spike proteins from variant spike proteins, including emerging variants of concern, and spike constructs containing point mutations within the receptor-binding domain. All pseudotyped lentiviral particles were replication-incompetent, used for single-cycle entry infectivity assays, and handled under approved institutional biosafety procedures at BSL-2 containment.

### Statement on AI use

Claude Opus 4.6–4.8 were used to assist in several areas of the codebase, including parsing input data files, evaluating existing models, templating the Transformer training loop, revising figure plotting code, and packaging antiGen as a Python library. While the code was edited and reviewed by AI tools, original implementations of the phylogenetic tree augmentation logic and the training loss function were written by the authors. All authors take full responsibility for the entire content of the manuscript, including any portions developed with the assistance of AI tools, and have reviewed and verified all text, code, figures, and results.

## Supplementary Material

Supplement 1

## Figures and Tables

**Figure 1 |:**
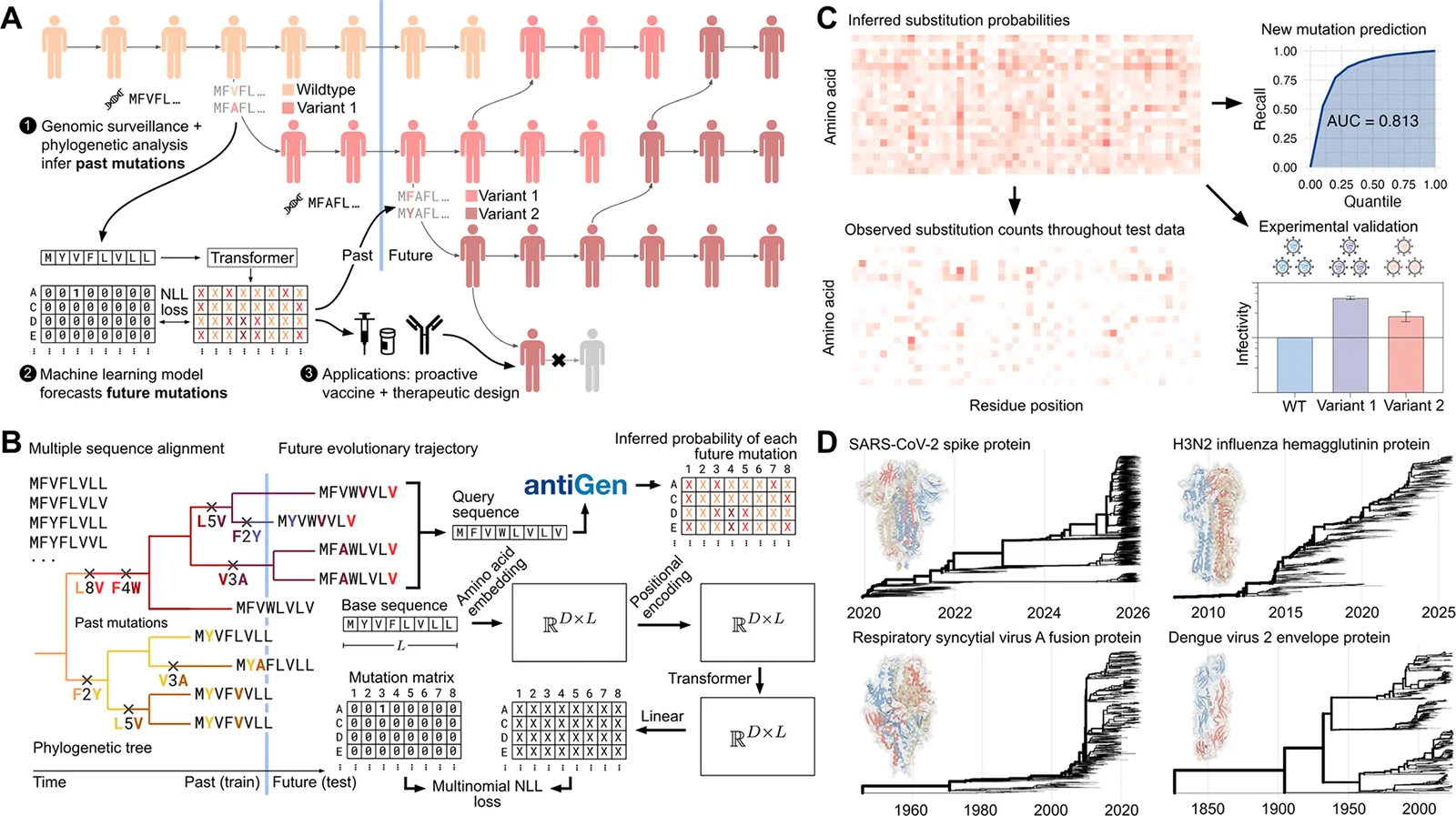
Overview of data, training, and evaluation for antiGen. (**A**) The goal of antiGen is to predict future mutations to viral proteins based on available genomic surveillance data at present. Mutation forecasts can be applied towards proactive vaccine and antibody design, potentially avoiding future outbreaks. (**B**) antiGen is a machine learning method trained on pandemic-scale phylogenetic trees annotated with inferred mutations along each branch. These mutation-annotated trees are partitioned into training and testing examples, each consisting of a past genotype together with the set of substitutions to have altered that genotype. antiGen then learns the probability of each possible mutation being the next to emerge, conditional on the starting sequence, using a likelihood-based training objective. (**C**) We evaluate antiGen several ways: its ability to predict the next mutation(s) to affect a given query sequence, to forecast which mutations will occur in future population-wide genomic surveillance data, and to propose mutations that remain compatible with pseudovirus infectivity in experimental validation. (**D**) We use antiGen to study the evolution of four key viral proteins: the SARS-CoV-2 spike protein (PDB 6X79), the influenza hemagglutinin protein (PDB 2YP2), the respiratory syncytial virus fusion protein (PDB 5TDL), and the dengue virus envelope protein (PDB 4UTC).

**Figure 2 |:**
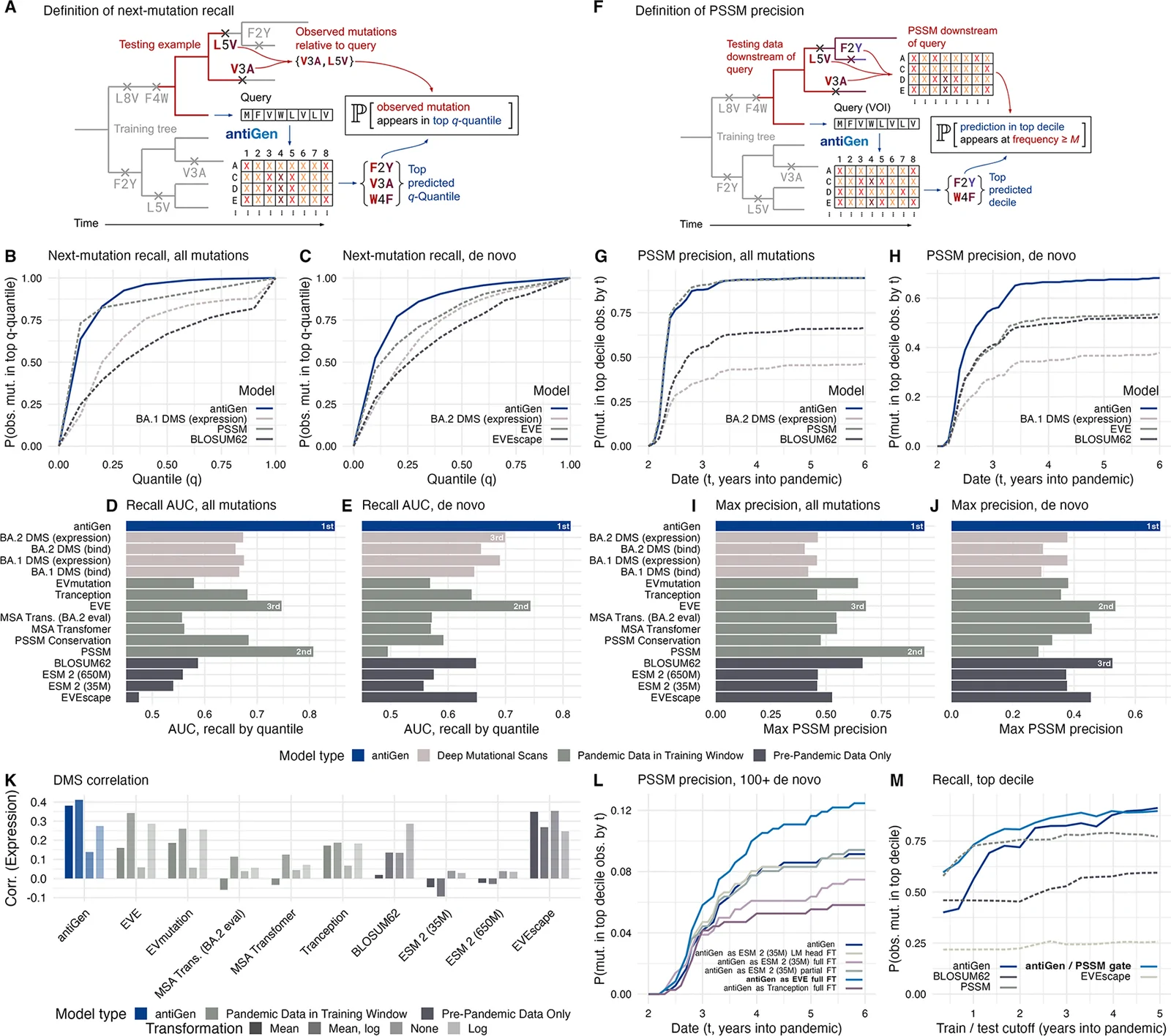
State-of-the-art performance for SARS-CoV-2 mutation forecasting. (**A**) Definition of next-mutation recall for a single testing example; overall recall is then calculated by averaging the recall across all testing examples. (**B, C**) Next-mutation-recall-by-quantile curves based on all mutations and *de novo* mutations (mutations never seen in training data), respectively. antiGen is shown together with the best-performing model from each of the following three categories: DMS experiments, models that train on genomic surveillance data up through the train-test cutoff, and pre-pandemic data alone. (**D, E**) Integral of the next-mutation-recall-by-quantile curve for all models considered in this study (excluding fine-tunes and gated hybrids), based on all mutations and *de novo* mutations, respectively. (**F**) Definition of PSSM precision. (**G, H**) PSSM precision as a function of the cutoff date within the testing window for which the PSSM downstream of the query is calculated, for all mutations and *de novo* mutations, respectively. (**I, J**) Maximum value of the PSSM-precision-by-cutoff-date curve for all models considered in this study (excluding fine-tunes and gated hybrids) based on all mutations and *de novo* mutations, respectively. (**K**) Correlation between model predictions and expression measurements for a BA.2 (Omicron) variant DMS (Starr et al., 2022). (**L**) Fine-tuning protein language models on the antiGen objective outperformed antiGen alone on some evaluations, such as prediction of *de novo* mutations downstream of BA.2 that reached a population frequency of 100 or greater. (**M**) Gating antiGen and the PSSM consistently outperformed either component, as well as pre-pandemic-data-only models, based on next-mutation recall for a variety of train-test cutoff dates.

**Figure 3 |:**
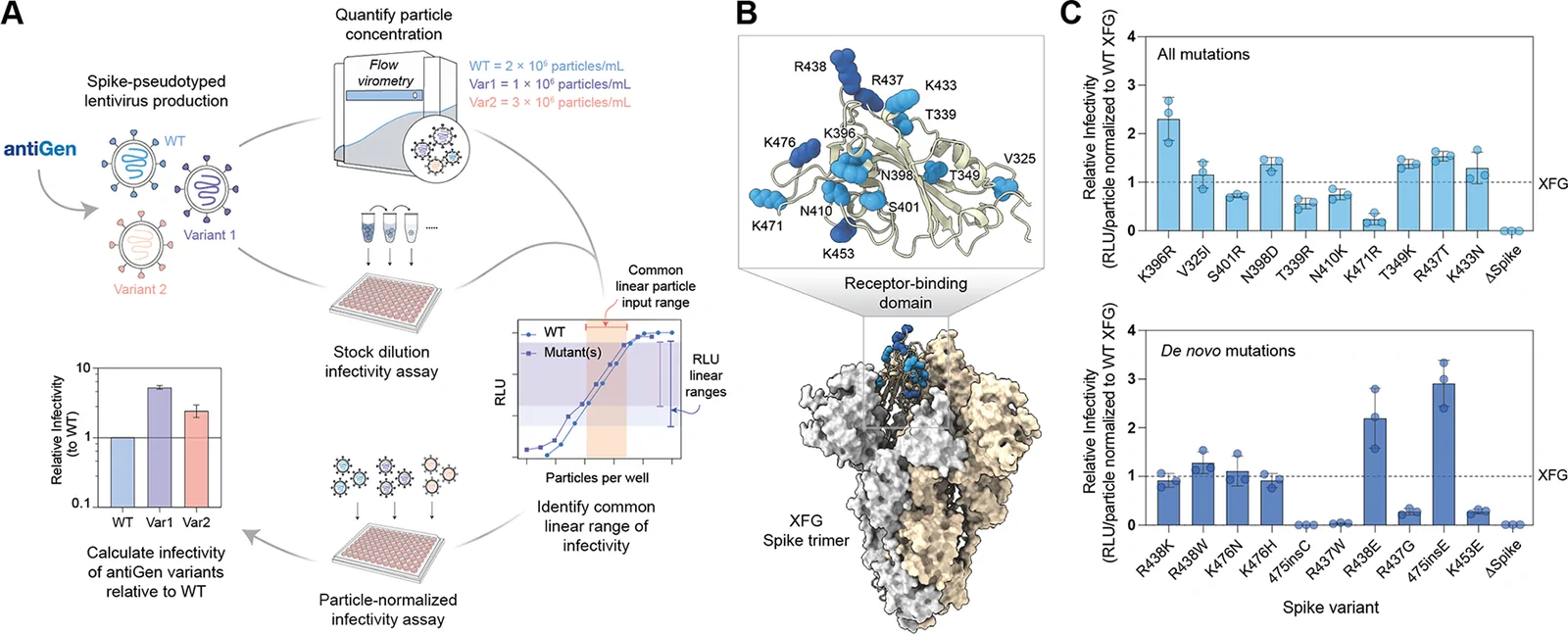
Infectivity of antiGen-predicted SARS-CoV-2 XFG spike variants. **(A)** Experimental workflow for testing antiGen-predicted spike variants. XFG wild-type (WT) and mutant spike-pseudotyped lentiviral particles were produced, quantified by flow virometry, and assayed for infectivity using a luciferase reporter assay following particle normalization. An initial stock-dilution infectivity assay was used to define a shared linear response range across all variants, after which variants were compared at matched particle inputs. **(B)** Structural mapping of antiGen-predicted mutations on an AlphaFold 3-predicted structure of the receptor-binding domain (RBD; residues 312–525) (top) of the SARS-CoV-2 spike trimer (bottom; PDB 8X4H) (Abramson et al., 2024). The ten highest-ranked predicted mutations across all candidate mutations are shown as light blue spheres; the ten highest-ranked *de novo* mutations are shown as dark blue spheres. **(C)** Particle-normalized relative infectivity of XFG spike variants carrying antiGen-predicted RBD mutations, categorized by mutation type as in (**B**). Infectivity was calculated as luciferase reporter signal (relative luminescence units; RLU) normalized to physical particle input determined by side scatter (SSC)-based flow virometry and expressed relative to XFG WT. Relative infectivity of a ΔSpike pseudovirus control, generated by omitting the spike-expression plasmid, is included as a spike-negative pseudovirus reference for background signal. Bars represent the mean of independent experiments; points indicate independent measurements; and error bars denote standard deviation. The dashed line denotes XFG WT infectivity.

**Figure 4 |:**
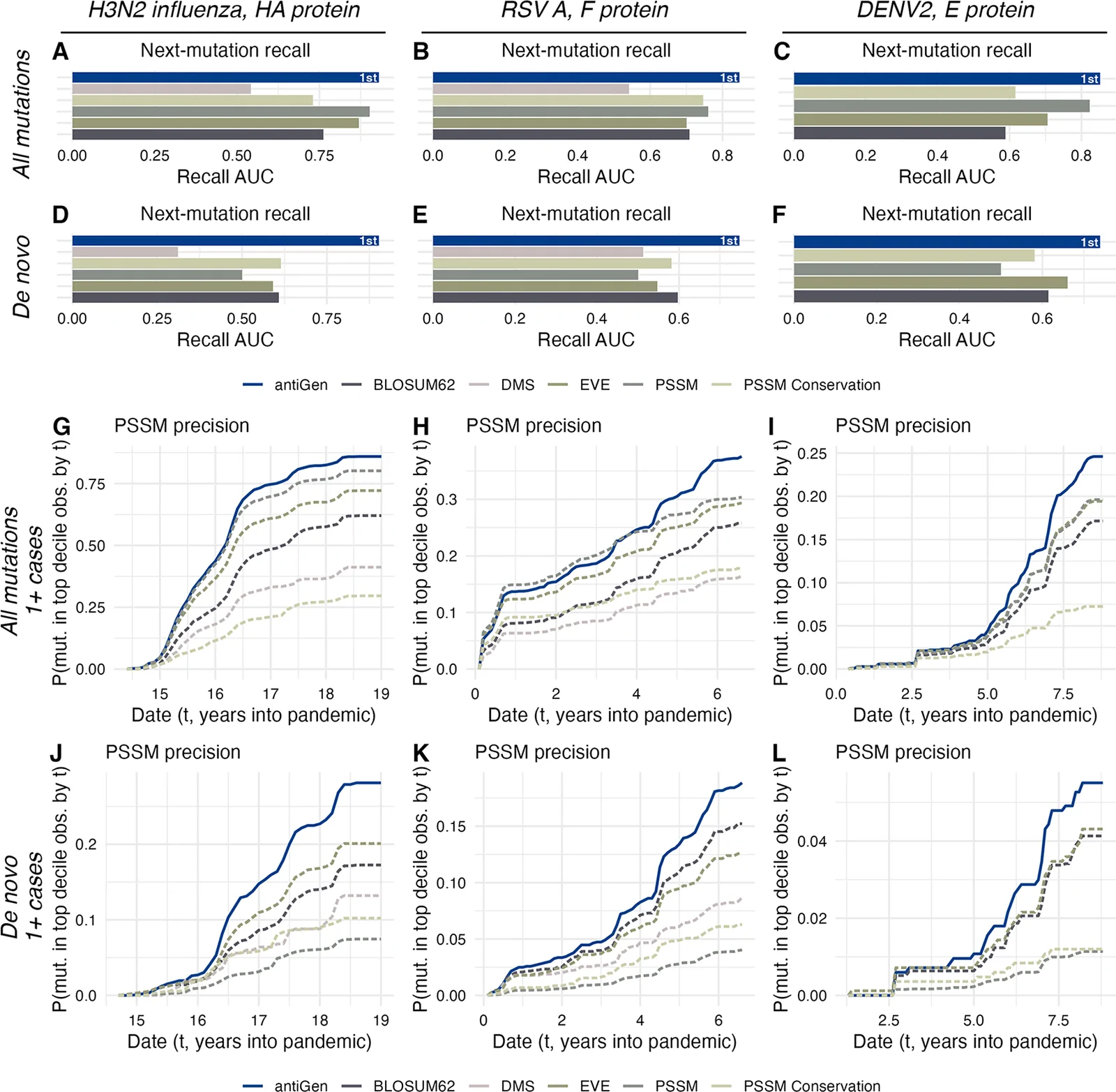
Mutation forecasting for endemic viruses. For H3N2 influenza (train-test cutoff: 2022), RSV A (train-test cutoff: 2020), and dengue virus 2 (train-test cutoff: 2019) we calculated the area under the next-mutation recall curve for all mutations (**A–C**) and *de novo* mutations (**D–F**). We calculated PSSM precision using the J subclade of influenza, the A.D.1.6 clade for RSV, and the 2II_F.1.1.2 minor lineage for dengue. We plotted PSSM precision curves (observed frequency cutoff ≥ 1) based on all mutations (**G–I**) and *de novo* mutations (**J–L**).

## Data Availability

Training data were assembled from public phylogenetic trees and sequences available via Nextstrain (Hadfield et al., 2018), LAPIS (Chen et al., 2023), and GISAID (Shu and McCauley, 2017; Wallau et al., 2023) as detailed in Methods. Sequences of the RBD mutants generated for pseudovirus variant production in this study, primer sequences used for cloning, and template vector information used for pseudovirus preparation are provided as **Data S1**. Amino acid sequences of the antibody heavy- and light-chain variable regions used in this study are provided as **Data S2**. Both data files are available to the public at https://doi.org/10.5281/zenodo.21481224.

## Code Availability

Model training, querying, and evaluation code, as well as the training data generation pipeline, are available to the public at https://github.com/evo-design/antiGen.
