# Supplementary material for: Forecasting viral evolution from phylogenetic trees
Source: bioRxiv. 2026 Sep 5:2026.08.27.741102. Preprint. [Version 1] doi: 10.64898/2026.08.27.741102 (PMC13684659; doi:10.64898/2026.08.27.741102)
Supplement: Supplementary file 1 [file NIHPP2026.08.27.741102v1-supplement-1.pdf]

bioRxiv preprint doi: https://doi.org/10.64898/2026.08.27.741102; this version posted September 5, 2026. The copyright holder for this preprint (which was not certified by peer review) is the author/funder, who has granted bioRxiv a license to display the preprint in perpetuity. It is made available under a CC-BY 4.0 International license.

	KP.3, binding	KP.3, expression	LP.8, binding	LP.8, expression
All mutations	0.0001004852	0.0004722464	0.00005804959	0.0006807268
De novo mutations	0.00861889	0.01658261	0.00865018	0.01861604

Table S1 | *p*-values for the count of mutations that increased pseudovirus infectivity in our experimental study, organized by DMS variant and measurement used for the background distribution.

bioRxiv preprint doi: https://doi.org/10.64898/2026.08.27.741102; this version posted September 5, 2026. The copyright holder for this preprint (which was not certified by peer review) is the author/funder, who has granted bioRxiv a license to display the preprint in perpetuity. It is made available under a CC-BY 4.0 International license.

Supplementary Figures

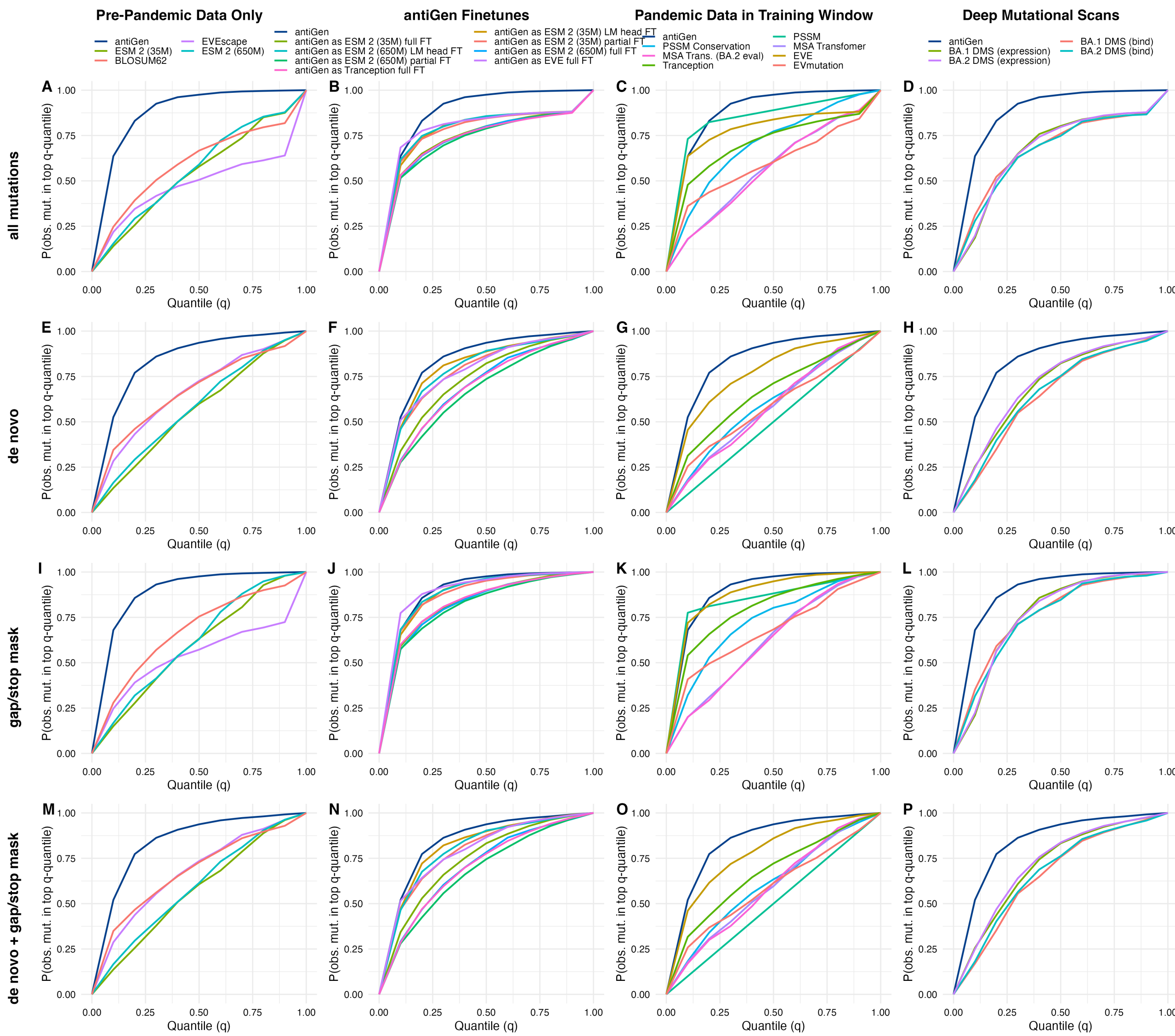

Figure S1 | Recall-by-quantile curves for the SARS-CoV-2 dataset based on prediction of (**A**)–(**D**) all mutations, (**E**)–(**H**) *de novo* mutations, (**I**)–(**L**) all mutations except for single-amino acid indels and mutations that introduce/remove premature stop codons, (**M**)–(**P**) *de novo* mutations excluding those involving gaps and stop codons. To avoid clutter, curves are grouped by model type as listed in the column headers.

bioRxiv preprint doi: https://doi.org/10.64898/2026.08.27.741102; this version posted September 5, 2026. The copyright holder for this preprint (which was not certified by peer review) is the author/funder, who has granted bioRxiv a license to display the preprint in perpetuity. It is made available under a CC-BY 4.0 International license.

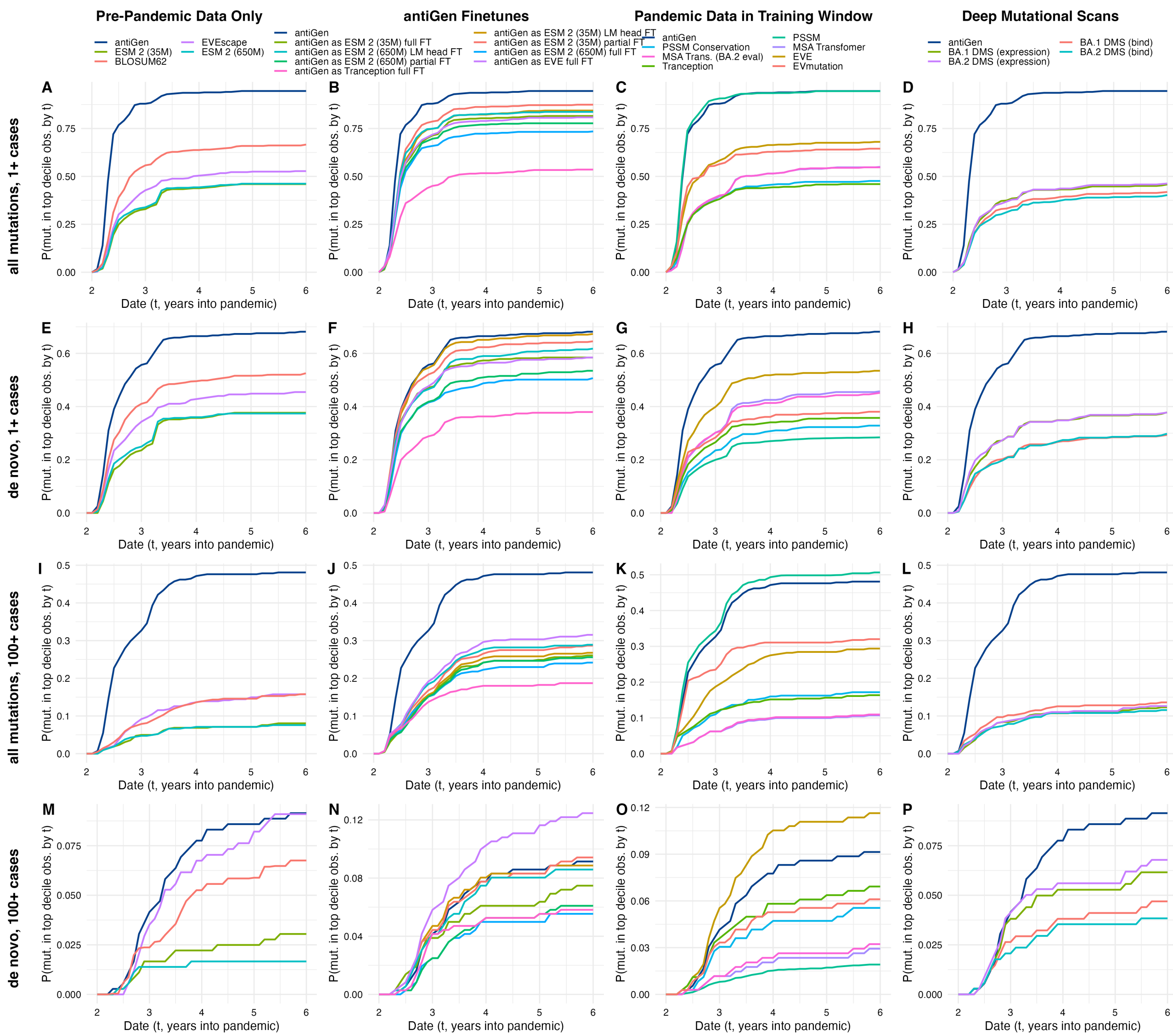

Figure S2 | PSSM-precision-by-cutoff-date curves for the SARS-CoV-2 dataset based on prediction of (**A**)–(**D**) all mutations, (**E**)–(**H**) *de novo* mutations, (**I**)–(**L**) all mutations except for single-amino acid indels and mutations that introduce/remove premature stop codons, (**M**)–(**P**) *de novo* mutations excluding those involving gaps and stop codons. To avoid clutter, curves are grouped by model type as listed in the column headers.

bioRxiv preprint doi: https://doi.org/10.64898/2026.08.27.741102; this version posted September 5, 2026. The copyright holder for this preprint (which was not certified by peer review) is the author/funder, who has granted bioRxiv a license to display the preprint in perpetuity. It is made available under a CC-BY 4.0 International license.

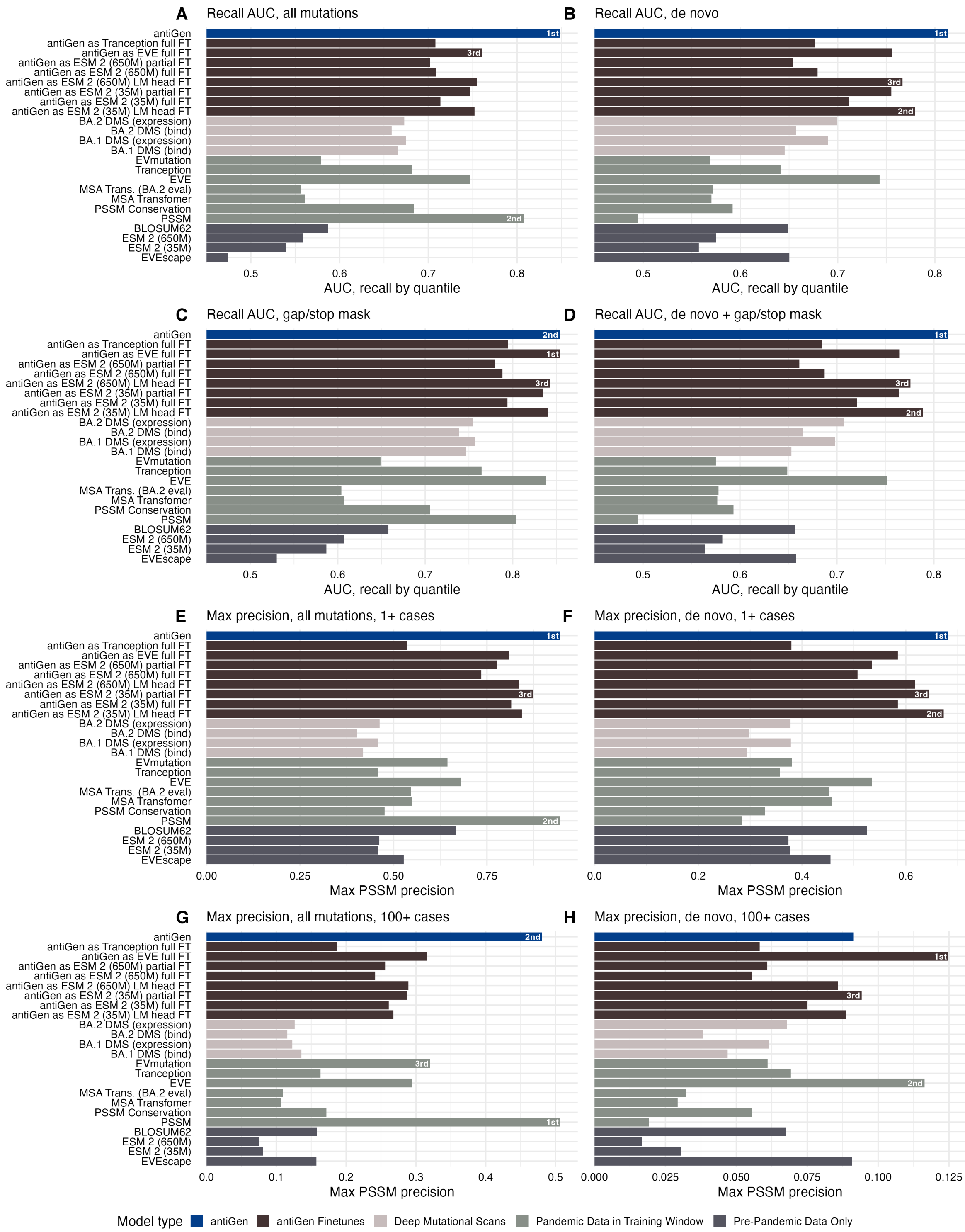

Figure S3 | AUCs of the next-mutation recall curves and maximum values of the PSSM precision curves shown in **Figure S1–S2**.

bioRxiv preprint doi: https://doi.org/10.64898/2026.08.27.741102; this version posted September 5, 2026. The copyright holder for this preprint (which was not certified by peer review) is the author/funder, who has granted bioRxiv a license to display the preprint in perpetuity. It is made available under a CC-BY 4.0 International license.

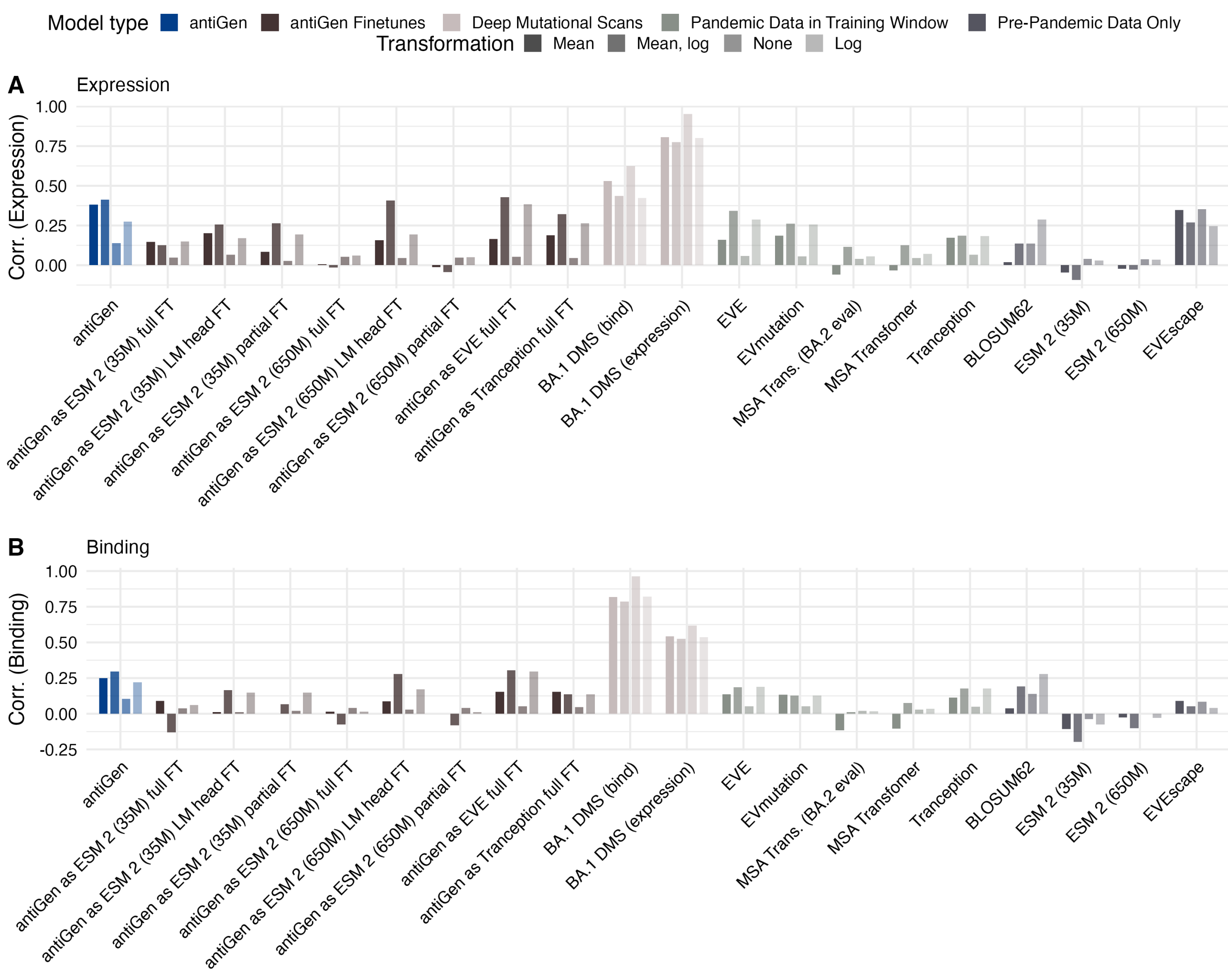

Figure S4 | BA.2 DMS correlation based on (**A**) expression and (**B**) binding measurements for all predictors, including a BA.1 DMS and fine-tuned models.

bioRxiv preprint doi: https://doi.org/10.64898/2026.08.27.741102; this version posted September 5, 2026. The copyright holder for this preprint (which was not certified by peer review) is the author/funder, who has granted bioRxiv a license to display the preprint in perpetuity. It is made available under a CC-BY 4.0 International license.

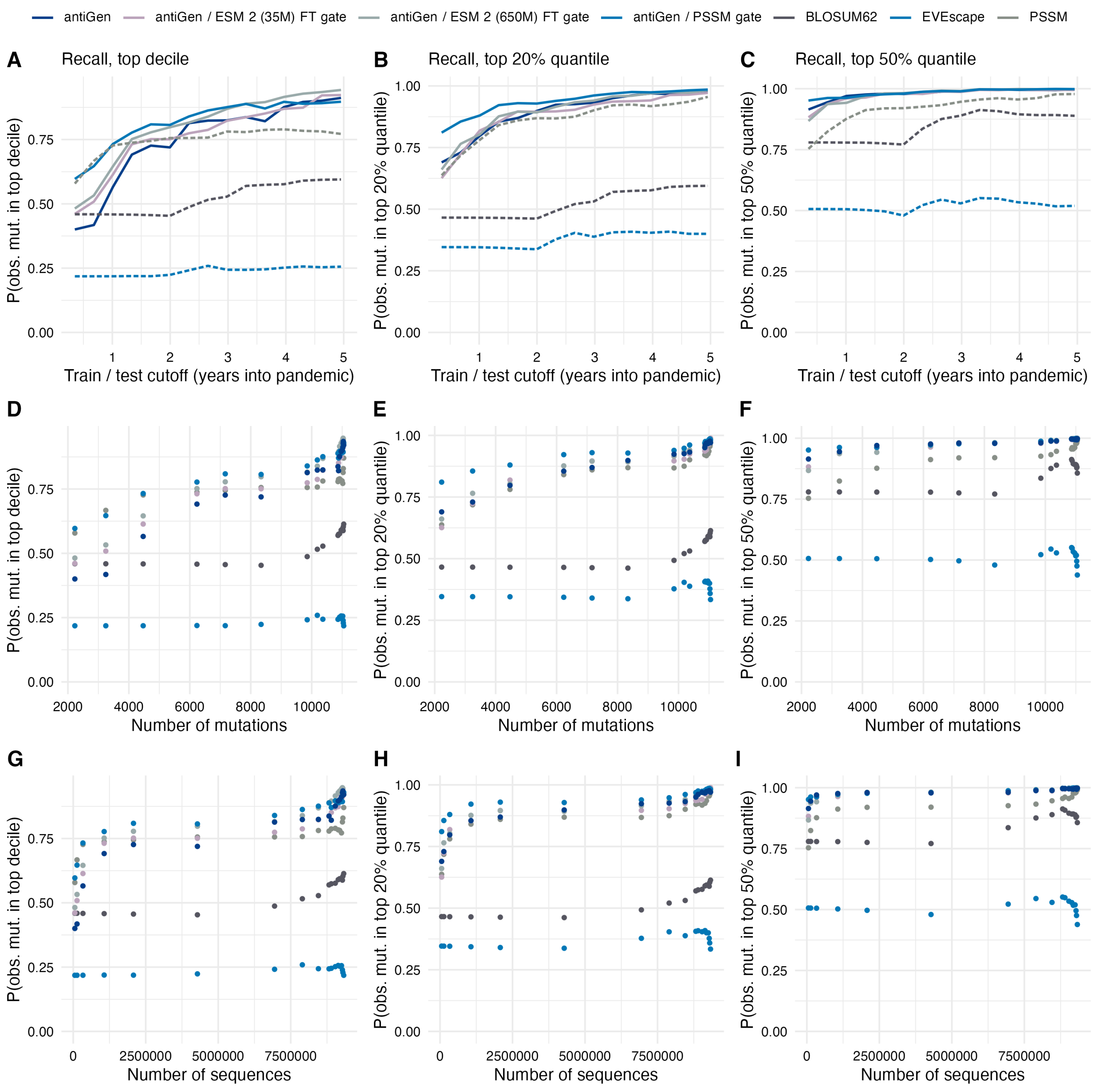

Figure S5 | Recall for gated models and pre-pandemic baselines for the (**A**, **D**, **G**) top decile, (**B**, **E**, **H**) top 20% quantile, and (**C**, **F**, **I**) top 50% quantile of predicted mutations for the SARS-CoV-2 dataset with various train-test cutoffs spanning the duration of the pandemic, organized by (**A–C**) train-test cutoff date, (**D–F**) number of mutations across all training examples, and (**G–I**) number of training sequences.

bioRxiv preprint doi: https://doi.org/10.64898/2026.08.27.741102; this version posted September 5, 2026. The copyright holder for this preprint (which was not certified by peer review) is the author/funder, who has granted bioRxiv a license to display the preprint in perpetuity. It is made available under a CC-BY 4.0 International license.

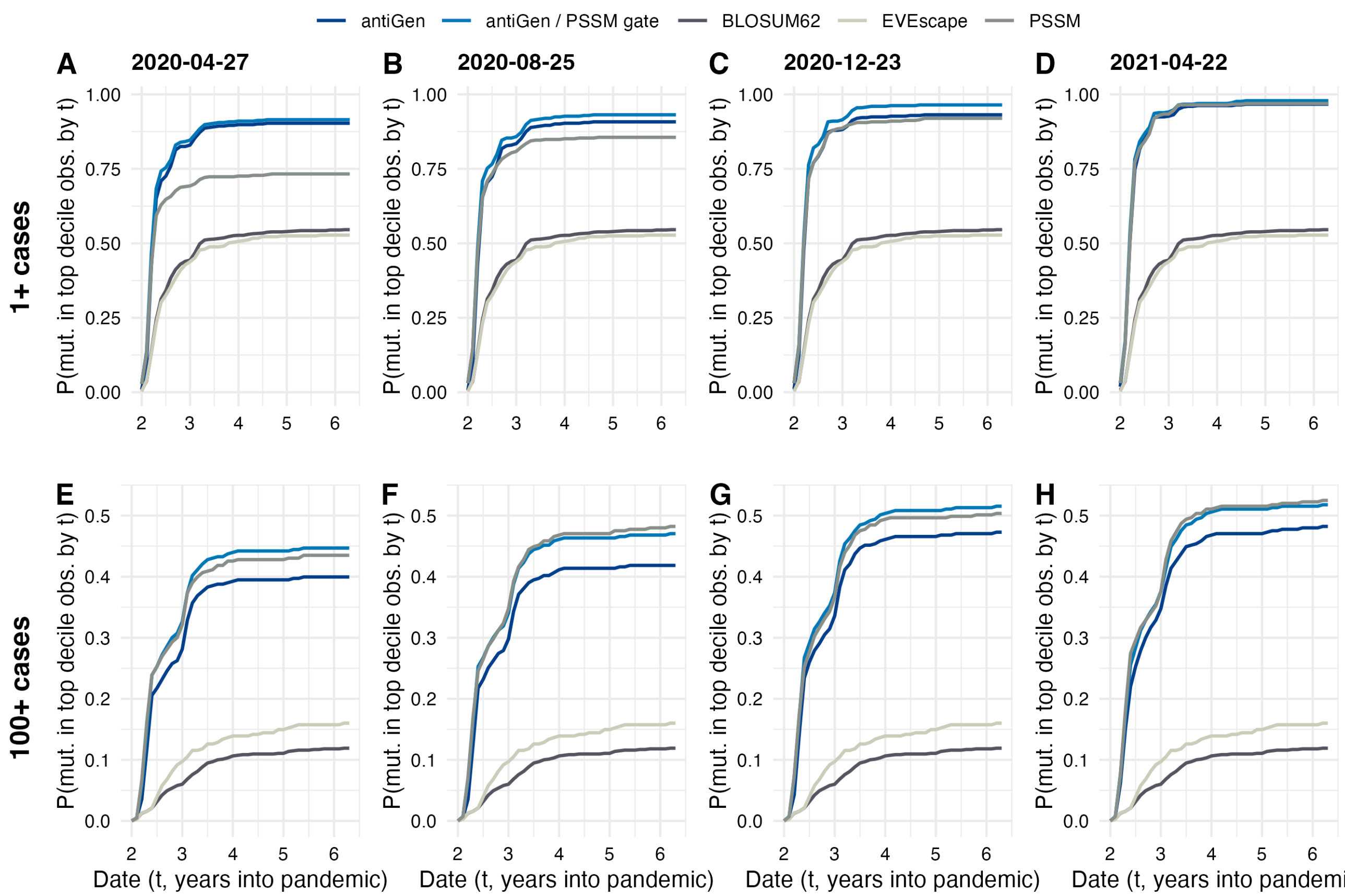

Figure S6 | PSSM precision downstream of BA.2 for the antiGen / PSSM gate, its two component models, and pre-pandemic baselines using various train-test cutoff dates prior to the emergence of BA.2. PSSM precision was calculated using a population frequency threshold of (**A–D**) 1 case and (**E–H**) 100 cases.

bioRxiv preprint doi: https://doi.org/10.64898/2026.08.27.741102; this version posted September 5, 2026. The copyright holder for this preprint (which was not certified by peer review) is the author/funder, who has granted bioRxiv a license to display the preprint in perpetuity. It is made available under a CC-BY 4.0 International license.

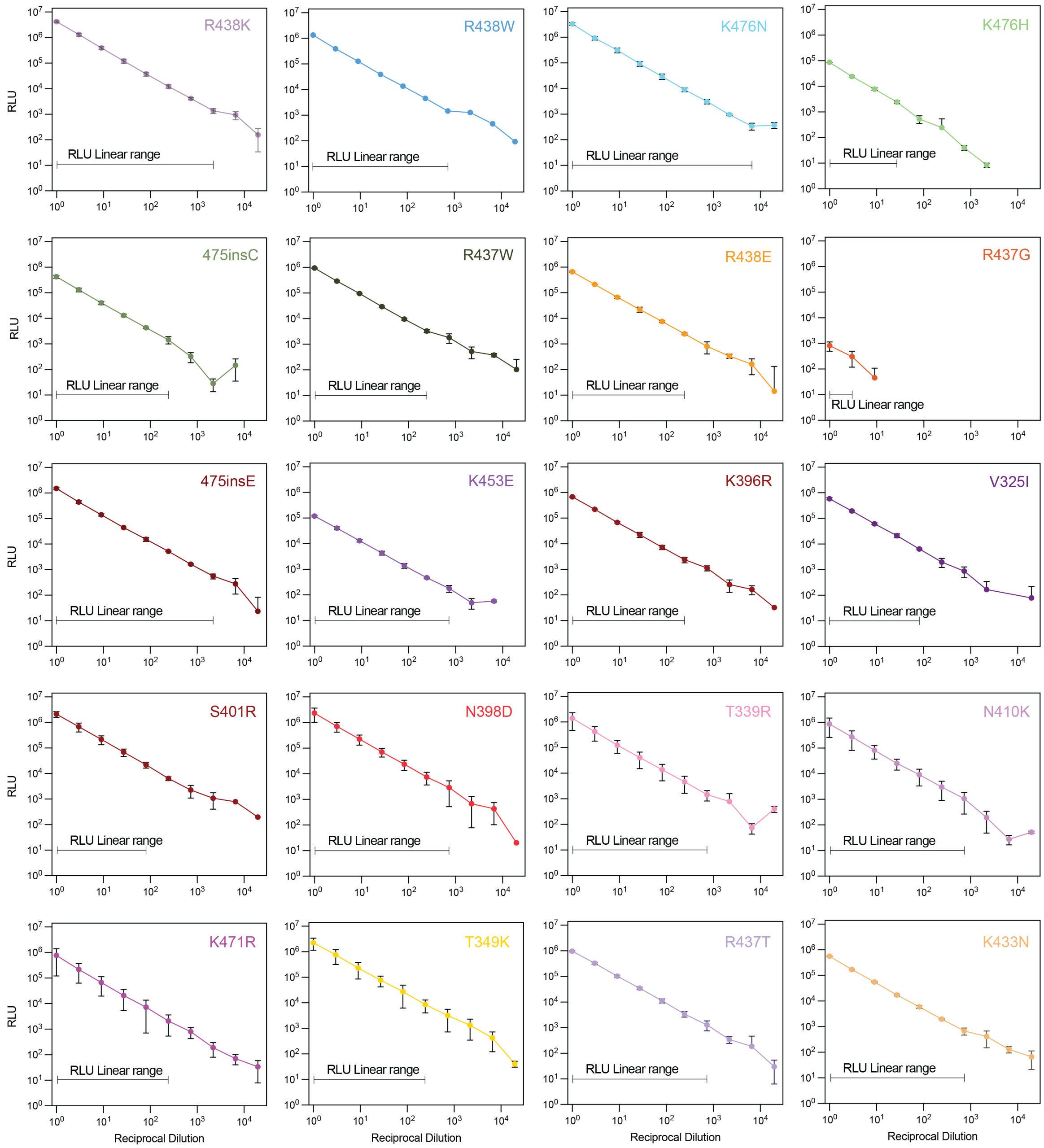

Figure S7 | **Serial-dilution infectivity profiling of antiGen-predicted SARS-CoV-2 XFG spike variants.** 10-point serial-dilution infectivity assays were performed for lentiviral pseudoviruses bearing XFG spike variants containing antiGen-predicted mutations. Luciferase reporter signal is plotted as RLU against reciprocal dilution for each pseudovirus stock. The dilution points over which reporter signal scaled proportionally with viral input are indicated on each plot. Points show mean values and error bars indicate standard deviation (n = 3).

bioRxiv preprint doi: https://doi.org/10.64898/2026.08.27.741102; this version posted September 5, 2026. The copyright holder for this preprint (which was not certified by peer review) is the author/funder, who has granted bioRxiv a license to display the preprint in perpetuity. It is made available under a CC-BY 4.0 International license.

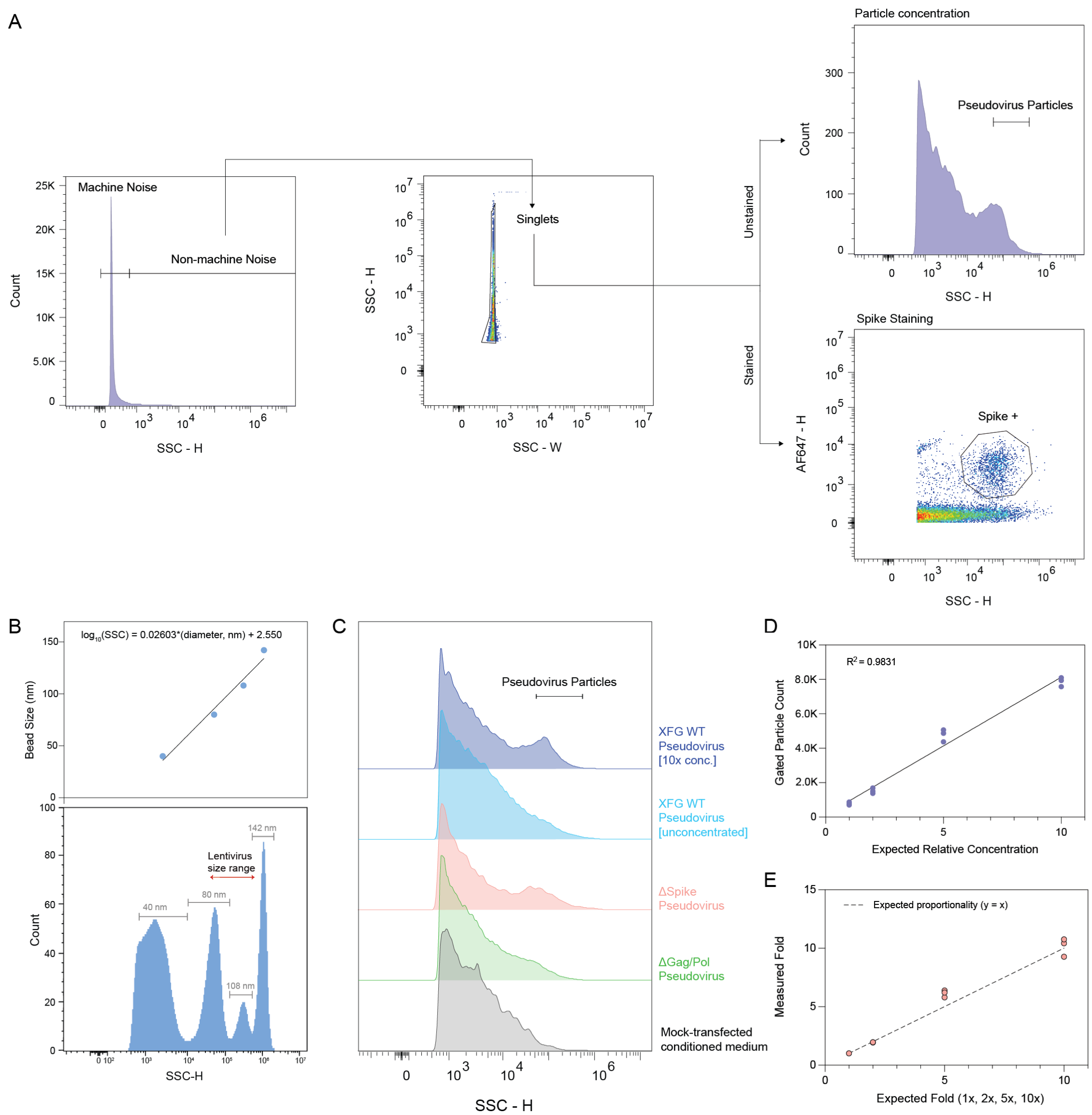

Figure S8 | **Establishment and validation of pseudovirus particle gating by flow virometry. (A)** Sequential CytoFLEX Nano gating strategy for pseudovirus particle identification. Events were separated from machine noise by SSC-H, singlets were selected using SSC-H and SSC-W, and pseudovirus-associated particles were gated by SSC-H; spike-positive events were identified by AF647 fluorescence after spike staining. **(B)** Calibration of SSC-H signal using size-standard beads. The relationship between bead diameter and SSC-H was used to define the approximate lentivirus size range for analysis. **(C)** Representative SSC-H distributions of mock-conditioned medium and pseudovirus preparations. WT pseudovirus after 10× concentration or pseudovirus lacking spike showed enrichment of a distinct SSC-H population relative to mock and pseudovirus production controls lacking Gag/Pol. **(D)** SSC-gated particle counts increased linearly with expected relative concentration in a WT pseudovirus dilution series (R^2 = 0.9831). **(E)** Measured fold changes approximately followed expected dilution factors, with underestimation at the highest concentration. These data validate the linear operating range of SSC-based particle enumeration used for subsequent particle quantification (n = 3).

bioRxiv preprint doi: https://doi.org/10.64898/2026.08.27.741102; this version posted September 5, 2026. The copyright holder for this preprint (which was not certified by peer review) is the author/funder, who has granted bioRxiv a license to display the preprint in perpetuity. It is made available under a CC-BY 4.0 International license.

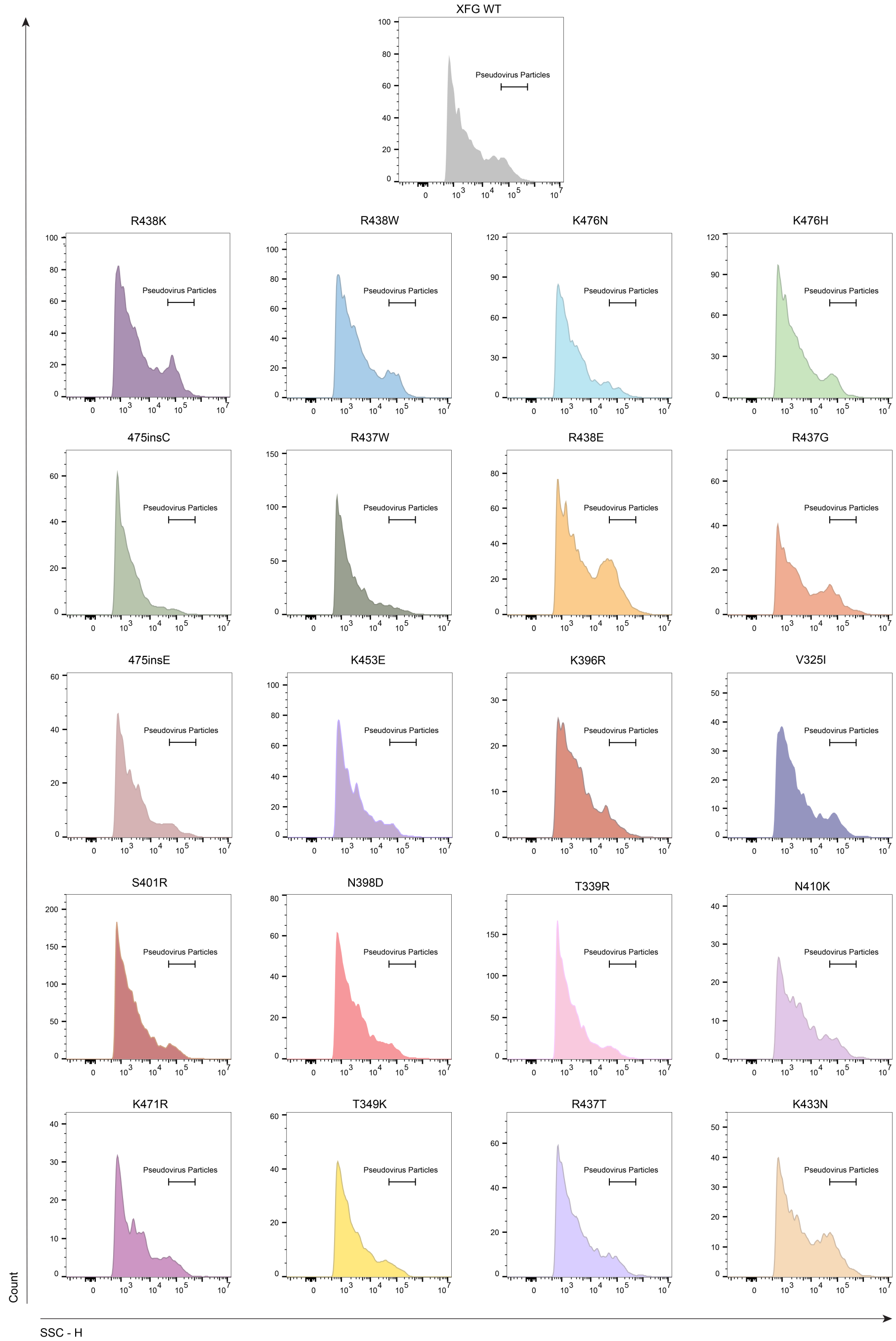

Figure S9 | **SSC-based particle gating for quantification of XFG spike-pseudotyped lentiviral preparations.** Representative SSC-H histograms of XFG WT and antiGen-predicted XFG spike variant pseudovirus preparations analyzed by flow virometry. The indicated SSC-H gate was applied consistently across all preparations to quantify pseudovirus particle count for downstream particle normalization. Histograms show the distribution of events after exclusion of machine noise and singlet gating, with particle counts derived from events falling within the defined pseudovirus particle gate. Representative histograms from one of two replicate acquisitions are shown, and stock particle concentrations were calculated as the mean of the two measurements ($n = 2$).

bioRxiv preprint doi: https://doi.org/10.64898/2026.08.27.741102; this version posted September 5, 2026. The copyright holder for this preprint (which was not certified by peer review) is the author/funder, who has granted bioRxiv a license to display the preprint in perpetuity. It is made available under a CC-BY 4.0 International license.

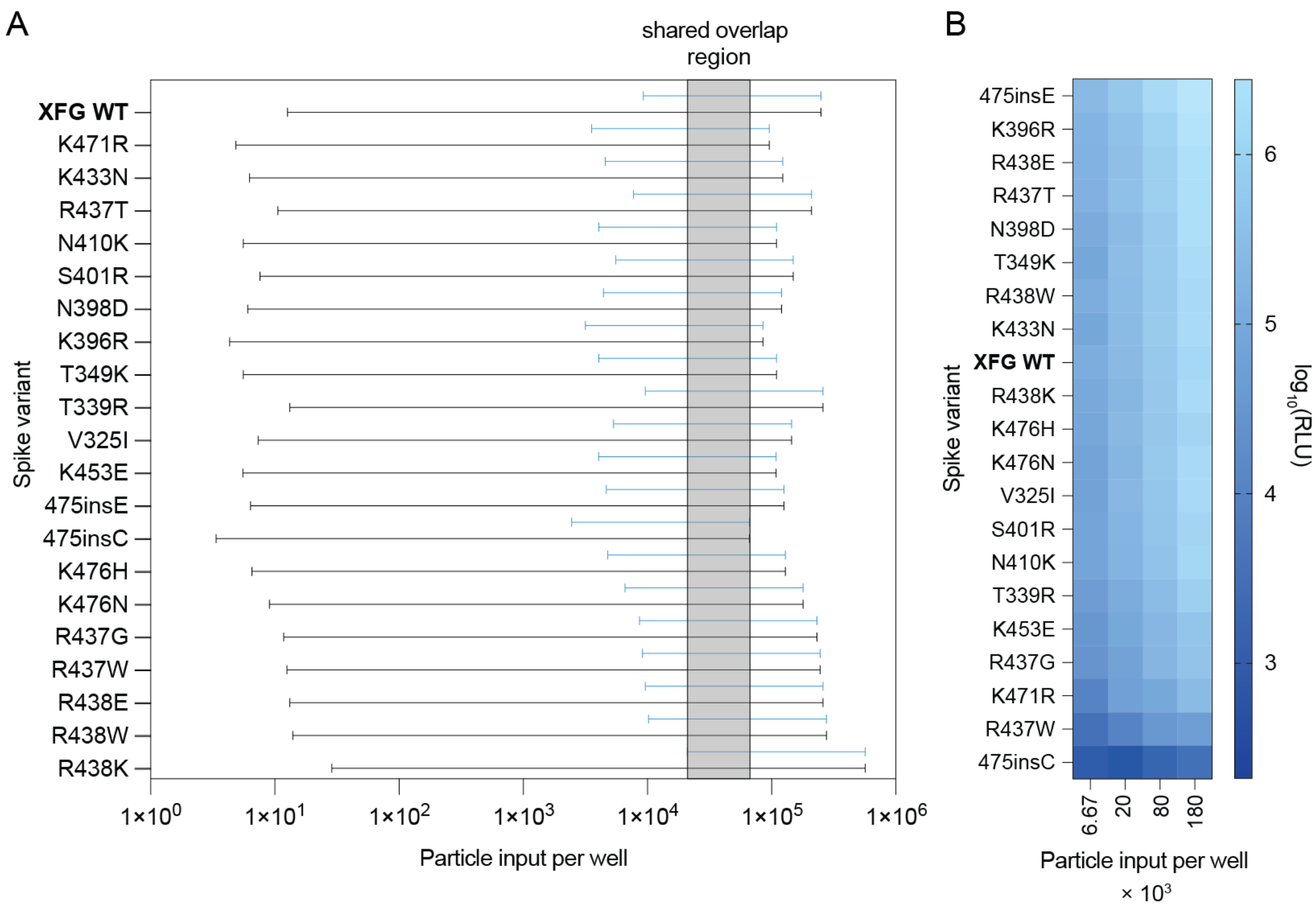

Figure S10 | **Defining a shared particle-input window for particle-normalized infectivity measurements. (A)** Construct-specific infectivity assay dilution ranges were converted into particle input per well using flow virometry-derived particle concentrations and the infection volume. Black horizontal lines indicate the full particle-input range sampled in the initial 10-point serial-dilution infectivity assay for each spike variant. The shaded region denotes the overlap of the particle-input ranges over which each pseudovirus variant exhibited an approximately linear relationship between particle input and RLU. Blue horizontal lines indicate the subset of the initial 10-point dilution series that fell within this common linear window; the corresponding shared particle-input range was subsequently used to design the four-point particle-normalized infectivity assay. **(B)** Heat map of luciferase reporter signal from the four-point particle-normalized infectivity assay across predetermined particle inputs.

bioRxiv preprint doi: https://doi.org/10.64898/2026.08.27.741102; this version posted September 5, 2026. The copyright holder for this preprint (which was not certified by peer review) is the author/funder, who has granted bioRxiv a license to display the preprint in perpetuity. It is made available under a CC-BY 4.0 International license.

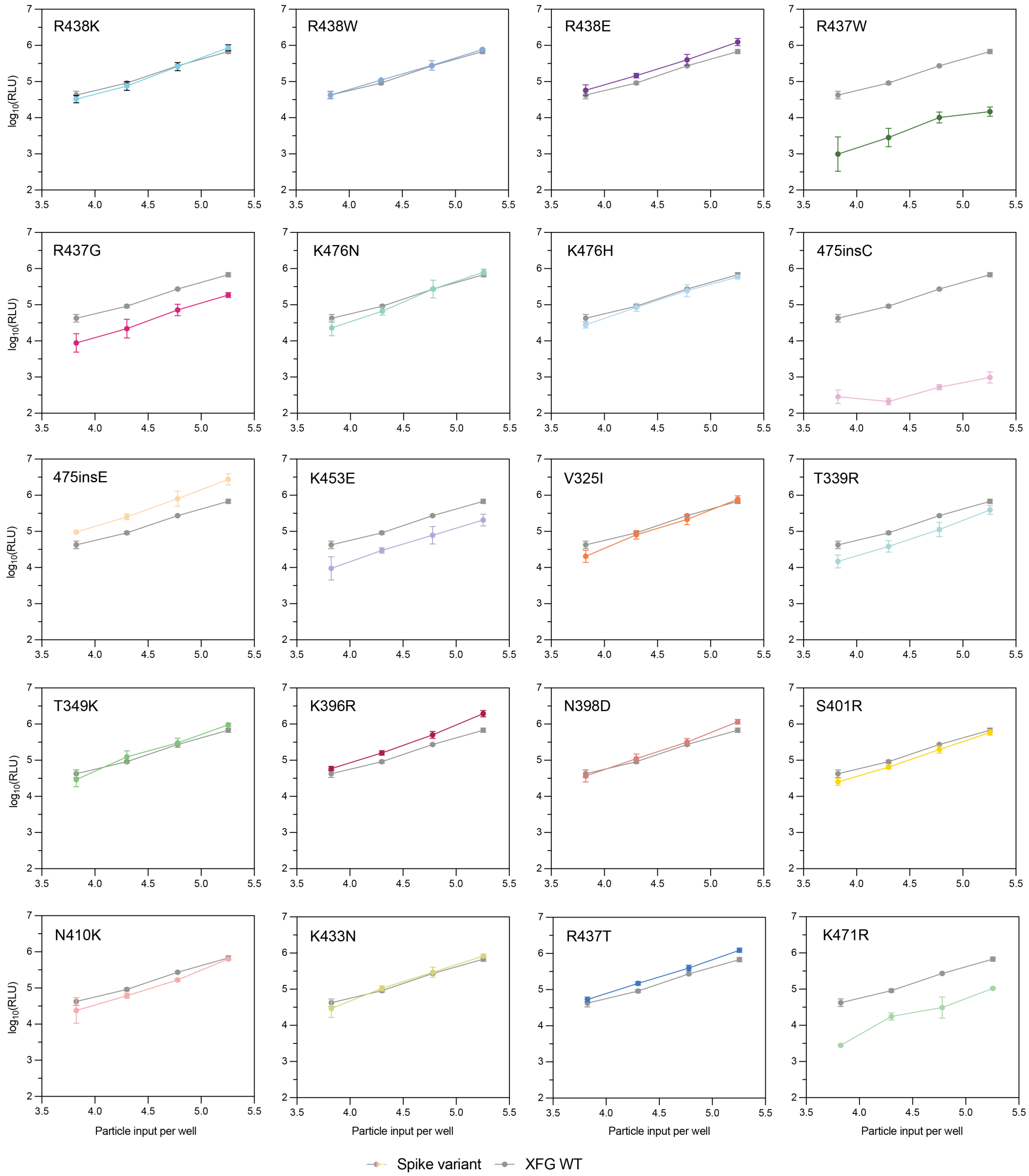

Figure S11 | **Particle-normalized infectivity assay of antiGen-predicted SARS-CoV-2 XFG spike variants.** Four-point, 3-fold serial-dilution infectivity assays were performed using pseudovirus inputs normalized by flow virometry-derived particle concentrations. Colored lines indicate each XFG spike variant, while gray lines show the XFG WT control. Particle inputs were selected such that the central dilution points fell within the shared linear operating window defined across variants, enabling comparison of infectivity at equivalent particle input. Points show mean values and error bars indicate standard deviation (n = 3).

bioRxiv preprint doi: https://doi.org/10.64898/2026.08.27.741102; this version posted September 5, 2026. The copyright holder for this preprint (which was not certified by peer review) is the author/funder, who has granted bioRxiv a license to display the preprint in perpetuity. It is made available under a CC-BY 4.0 International license.

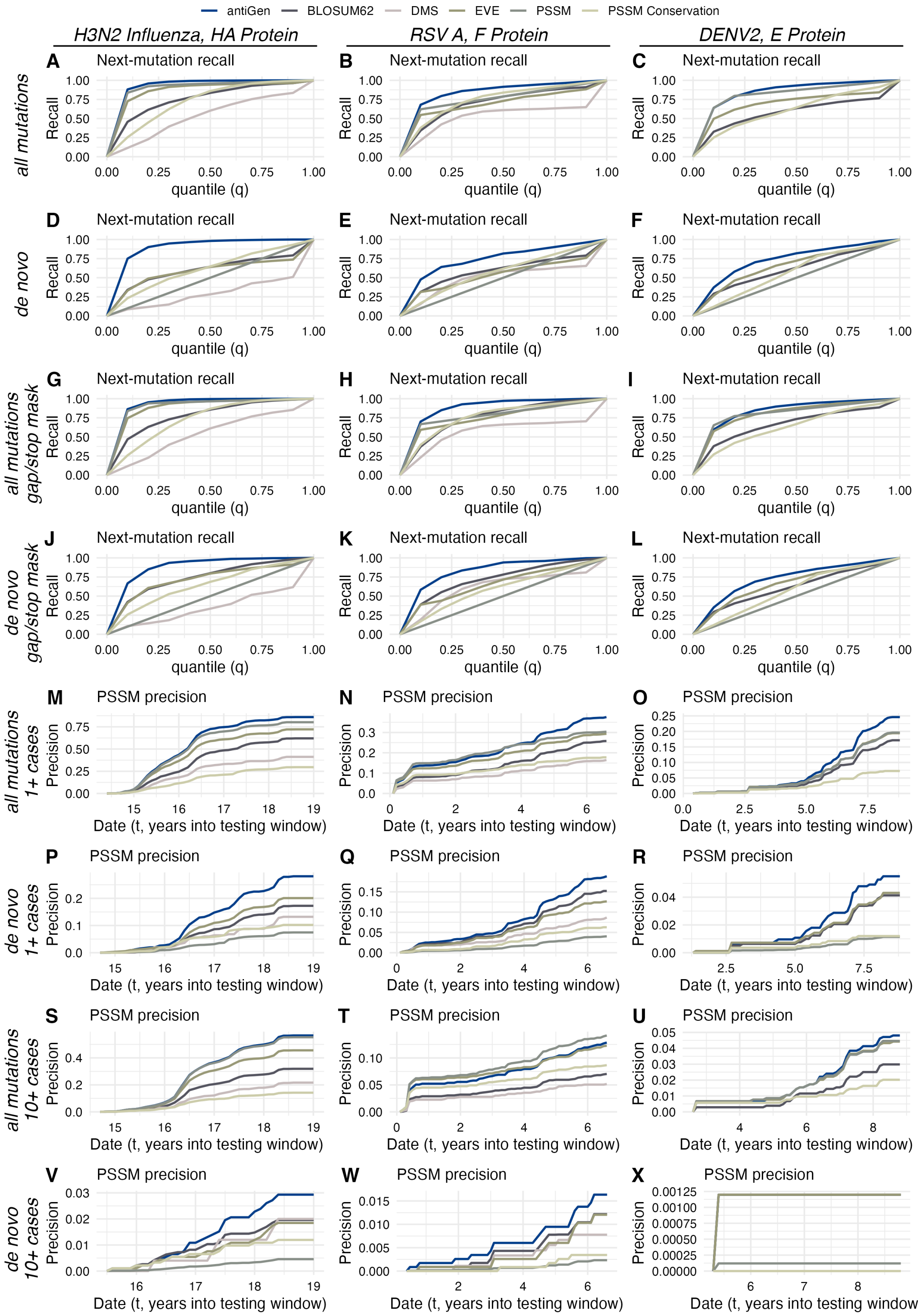

Figure S12 | Full recall-by-quantile curves broken down by all versus *de novo* mutations and with versus without a mask for gap and stop codons (**A–L**), and PSSM-precision-by-cutoff-date curves broken down by all versus *de novo* mutations and for observed frequency 1 versus 10 (**M–X**) for influenza, RSV, and dengue.

bioRxiv preprint doi: https://doi.org/10.64898/2026.08.27.741102; this version posted September 5, 2026. The copyright holder for this preprint (which was not certified by peer review) is the author/funder, who has granted bioRxiv a license to display the preprint in perpetuity. It is made available under a CC-BY 4.0 International license.

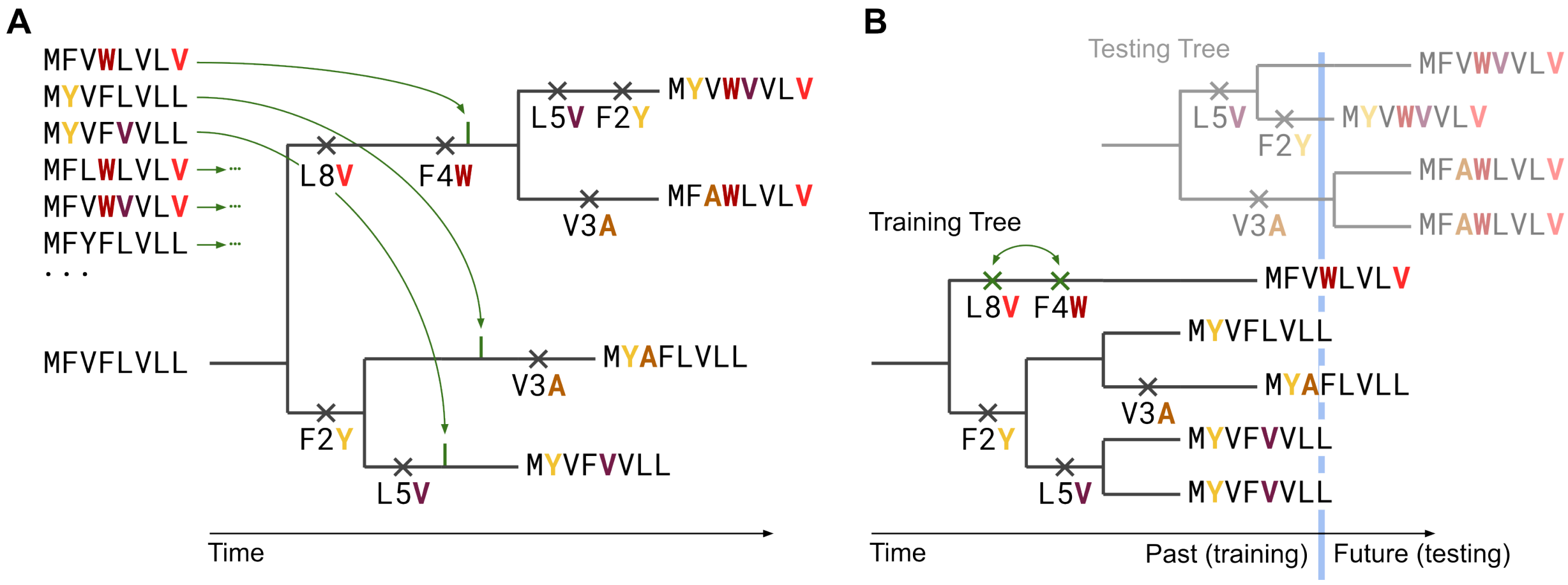

Figure S13 | Core ideas behind the phylogenetic tree augmentation algorithm used to produce training data for antiGen. (**A**) Amino acid sequences in a large multiple sequence alignment are iteratively attached to the phylogenetic tree at the location that minimizes the number of new substitutions introduced, reordering existing mutations on branches if necessary. (**B**) For each branch with multiple mutations, the order of mutations is randomized. Then, the subtree(s) ancestral to a user-specified cutoff date are pruned to separate the testing tree(s) from the training tree. Note that the actual training data generation algorithm compresses phylogenetic trees into mutation trees for improved performance; the visualization here shows full coalescent histories to provide intuition.

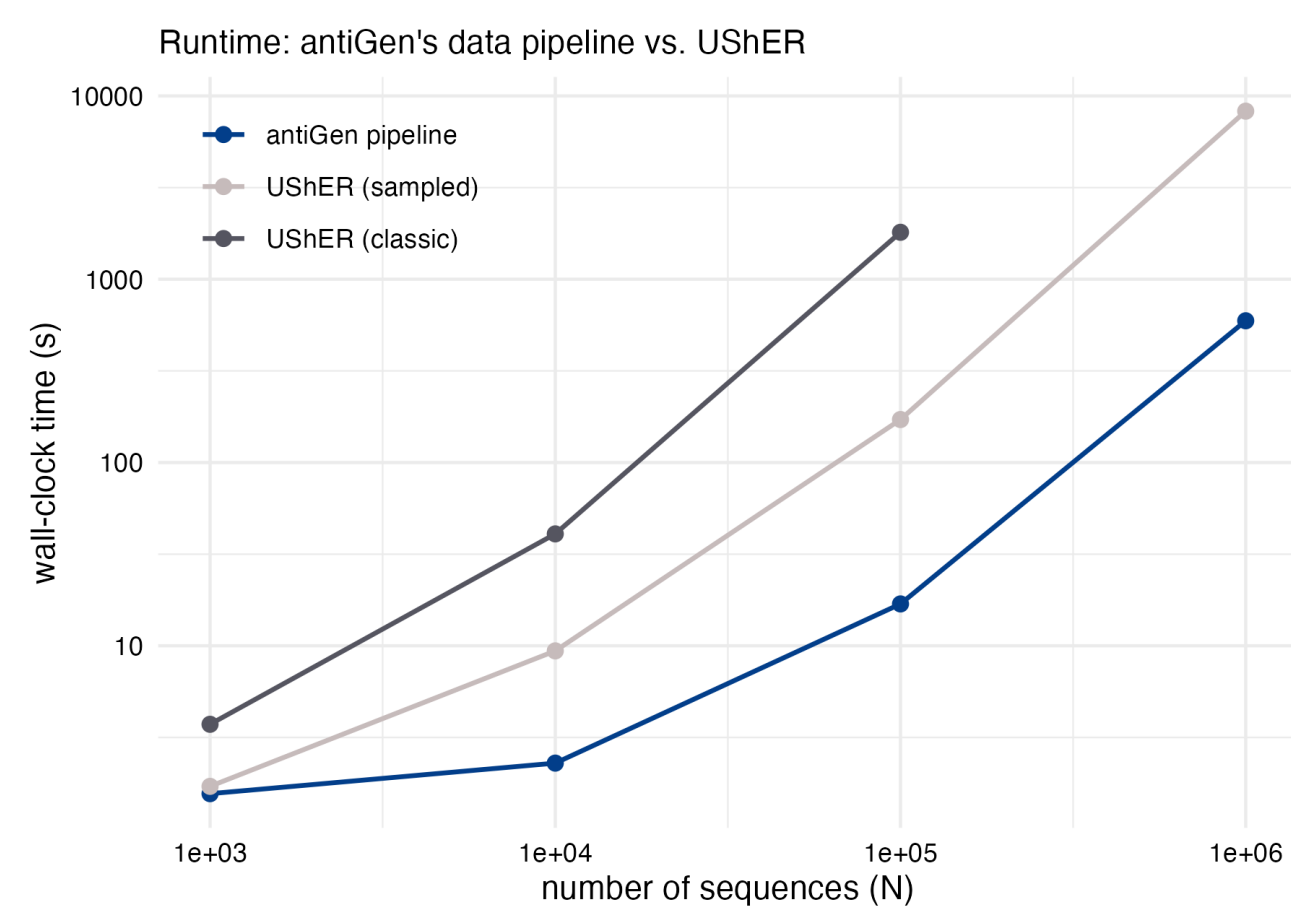

Figure S14 | Runtime comparison between antiGen's data generation pipeline and UShER (Turakhia et al., 2021) run two ways: "sampled," the latest CPU parallel method (which also includes a subtree-prune-regraft tree refinement step), and "classic" (original algorithm). The data used to benchmark both tools are the Nextstrain SARS-CoV-2 tree used throughout this paper, together with sequences obtained from the University of California, Santa Cruz SARS-CoV-2 genome browser (Casper et al., 2026). All genomes were filtered to the spike protein region as an analog to the tree augmentation use case in this paper. The "classic" mode is only shown for up to 100,000 sequences due to long runtimes at greater scale.

bioRxiv preprint doi: https://doi.org/10.64898/2026.08.27.741102; this version posted September 5, 2026. The copyright holder for this preprint (which was not certified by peer review) is the author/funder, who has granted bioRxiv a license to display the preprint in perpetuity. It is made available under a CC-BY 4.0 International license.

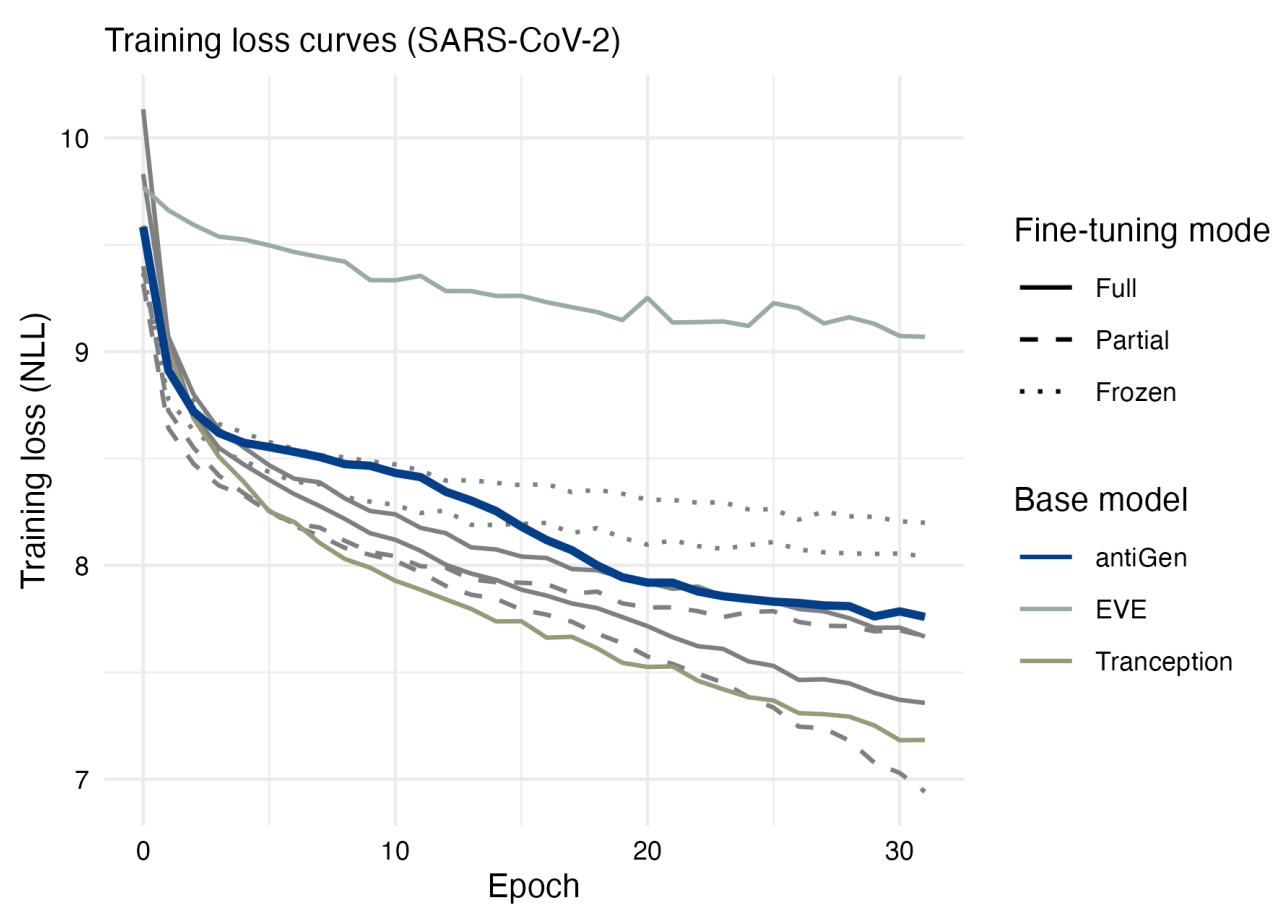

Figure S15 | Training loss on the SARS-CoV-2 dataset for antiGen, as well as EVE, ESM-2, and Tranception fine-tuned on the antiGen data and loss.

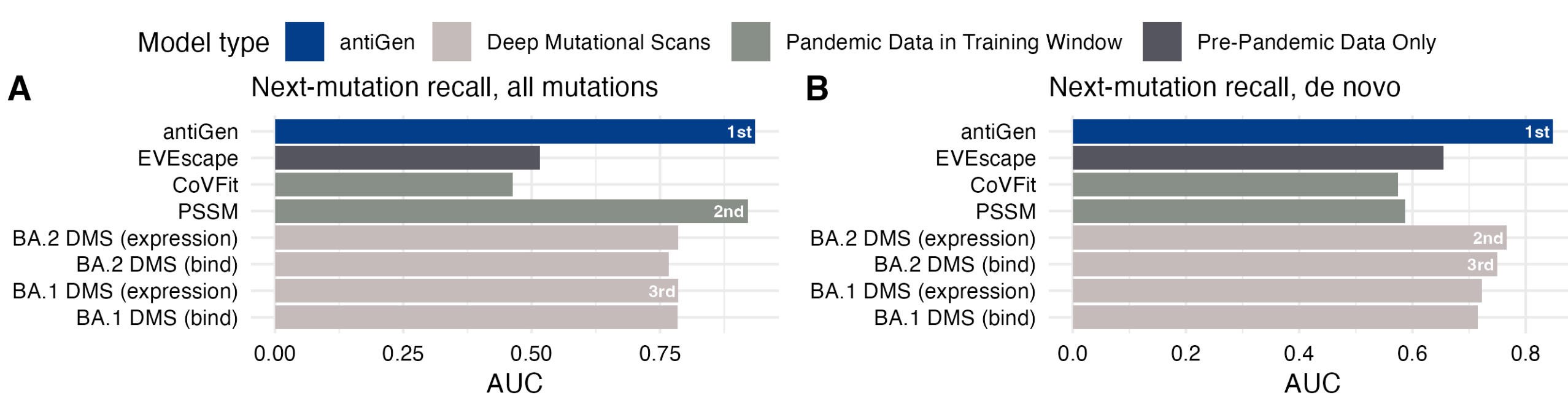

Figure S16 | Recall AUC for CoVFit, antiGen with the same training window as CoVFit, and various baseline models, subset by (**A**) all mutations and (**B**) *de novo* mutations.

bioRxiv preprint doi: https://doi.org/10.64898/2026.08.27.741102; this version posted September 5, 2026. The copyright holder for this preprint (which was not certified by peer review) is the author/funder, who has granted bioRxiv a license to display the preprint in perpetuity. It is made available under a CC-BY 4.0 International license.

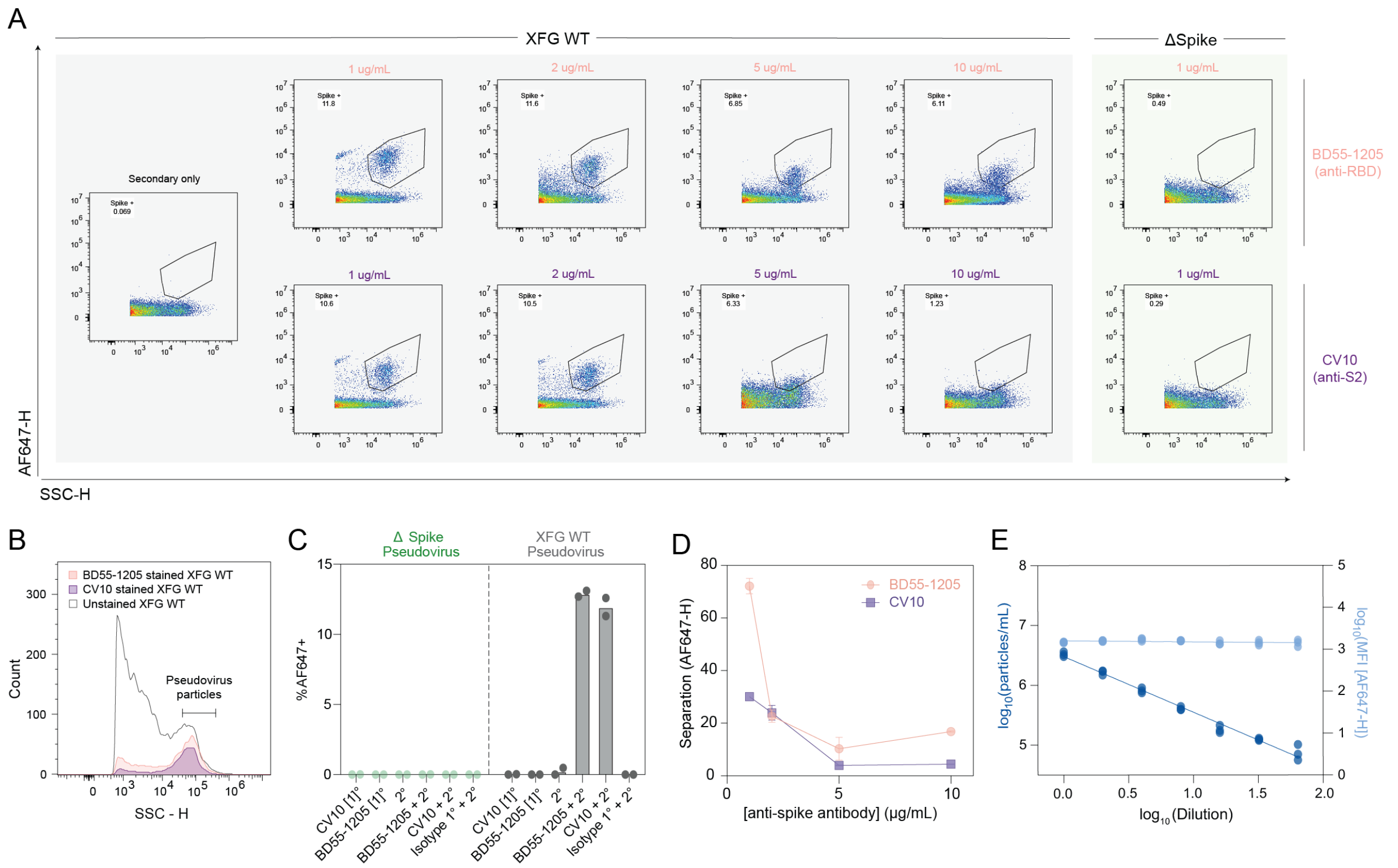

Figure S17 | **Optimization of flow virometric detection of intact SARS-CoV-2 spike on pseudotyped lentivirus particles.** **(A)** Flow virometry detection of XFG WT spike-pseudotyped particles stained with titrated amounts of BD55-1205 anti-RBD or CV10 anti-S2 primary antibodies, followed by AF647-conjugated secondary antibody. Secondary-only and ΔSpike pseudovirus controls were used to define background staining and spike-positive gates. **(B)** SSC-H distributions of unstained, BD55-1205-stained, and CV10-stained XFG WT pseudovirus preparations. Spike-positive particles identified by S2 or RBD staining localized predominantly within the predefined pseudovirus particle gate, supporting enrichment of spike-bearing particles within the SSC-defined population. **(C)** Validation of the spike-positive gate using primary-only, secondary-only, and isotype control conditions. AF647+ events within the defined gate were observed only when both BD55-1205 or CV10 primary and AF647-conjugated secondary antibodies were applied to XFG WT particles, and not to ΔSpike particles, confirming that the gate specifically reports spike-dependent, antibody-specific staining. **(D)** Primary antibody titration for BD55-1205 and CV10 staining of XFG WT pseudovirus particles. Staining performance was evaluated using the stain index, a measure of separation between AF647-positive and AF647-negative particle populations, to identify the primary antibody concentration providing the greatest separation from background ($n = 2$). **(E)** Effect of pseudovirus dilution on particle recovery and spike-staining intensity. SSC-gated particle concentration decreased proportionally with dilution, whereas the AF647-H median fluorescence intensity of the antibody-positive population remained stable across the tested range. The stability of AF647 fluorescence intensity across serial dilutions indicates that fluorescence measurements were not substantially influenced by particle concentration within the validated operating range, consistent with single-particle detection. Together with the localization of antibody-positive particles within the predefined SSC gate and the specificity controls, these data support the use of SSC-based particle counts as a proxy for pseudovirus particle input in subsequent normalization experiments ($n = 3$).

bioRxiv preprint doi: https://doi.org/10.64898/2026.08.27.741102; this version posted September 5, 2026. The copyright holder for this preprint (which was not certified by peer review) is the author/funder, who has granted bioRxiv a license to display the preprint in perpetuity. It is made available under a CC-BY 4.0 International license.

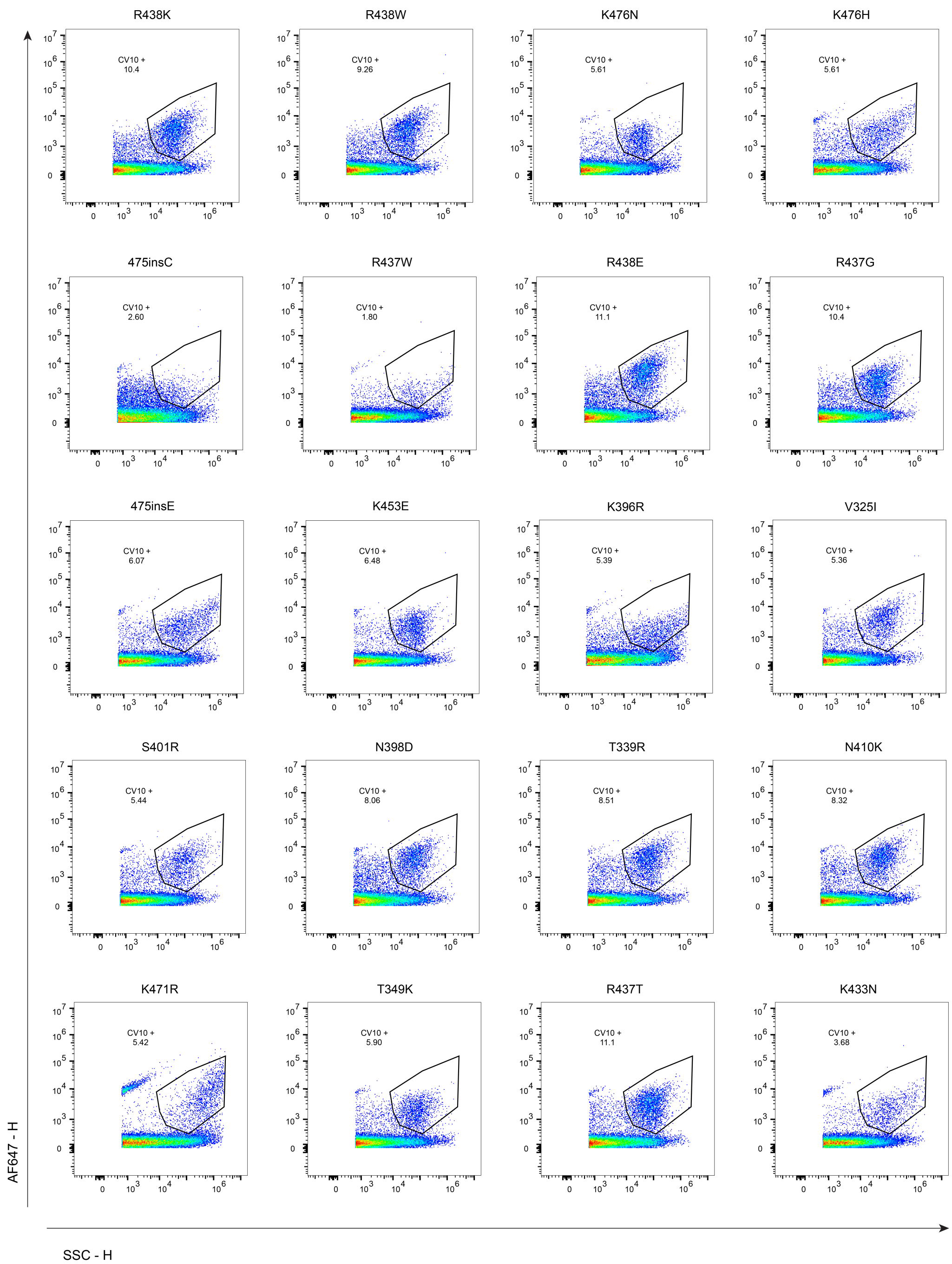

Figure S18 | **Anti-S2 CV10 staining of spike-bearing particles across XFG spike variants.** Representative flow-virometry plots of 10× concentrated pseudovirus preparations stained with CV10, an S2-specific monoclonal antibody, followed by AF647-conjugated goat anti-human IgG secondary antibody detection. AF647-positive populations were identified using a common fluorescence gate established from secondary-only controls. The percentage of CV10-positive events is indicated for XFG WT and each antiGen-predicted spike variant (n = 2).

bioRxiv preprint doi: https://doi.org/10.64898/2026.08.27.741102; this version posted September 5, 2026. The copyright holder for this preprint (which was not certified by peer review) is the author/funder, who has granted bioRxiv a license to display the preprint in perpetuity. It is made available under a CC-BY 4.0 International license.

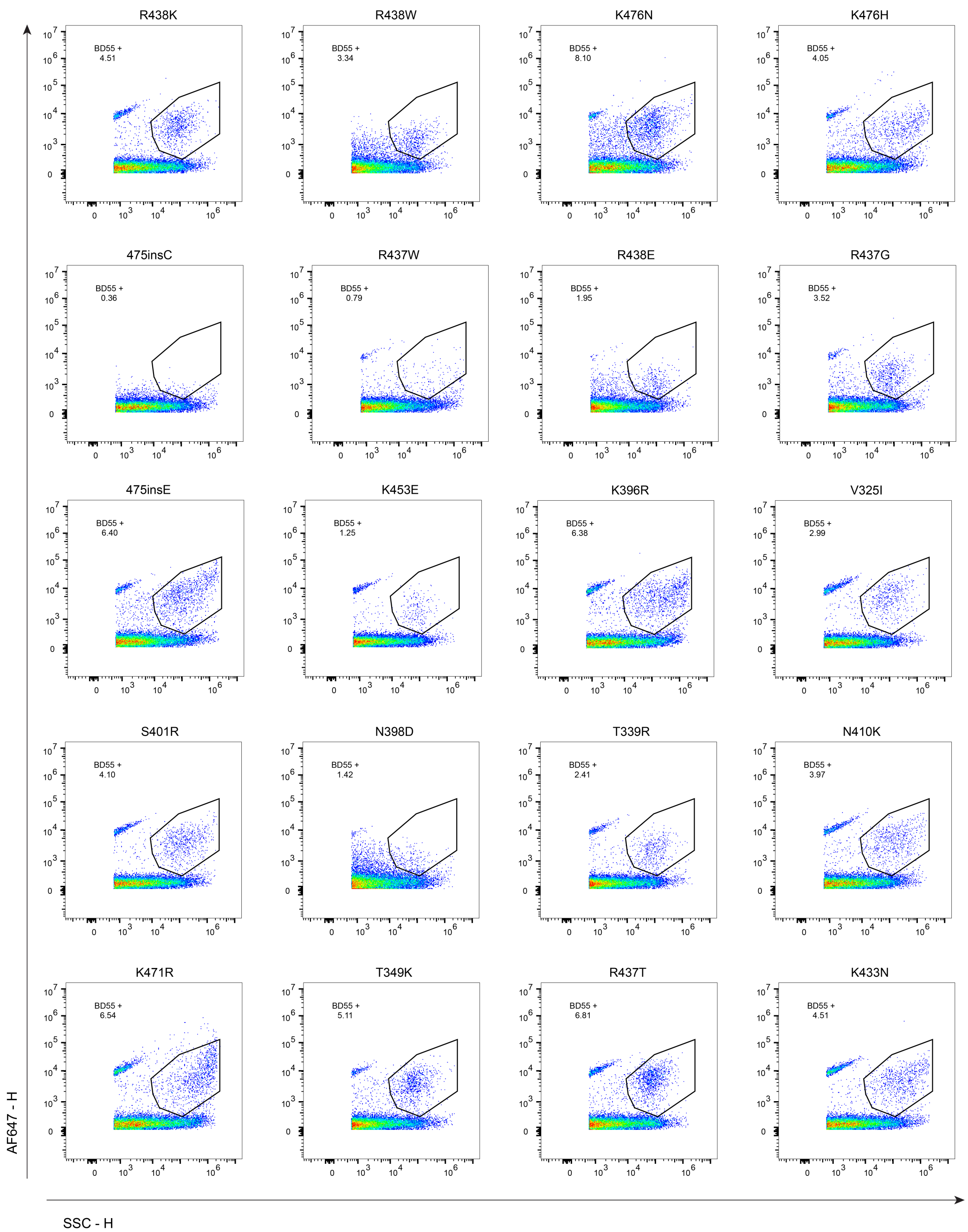

Figure S19 | **Anti-RBD BD55-1205 staining of spike-bearing particles across XFG spike variants.** Representative flow-virometry plots of 10× concentrated pseudovirus preparations stained with BD55-1205, an anti-RBD monoclonal antibody, followed by AF647-conjugated goat anti-human IgG secondary antibody detection. AF647-positive populations were identified using a common fluorescence gate established from secondary-only controls. The percentage of BD55-1205-positive events is indicated for each spike variant (n = 2).
